# Combining Web-Based Gamification and Physical Nudges With an App (MoveMore) to Promote Walking Breaks and Reduce Sedentary Behavior of Office Workers: Field Study

**DOI:** 10.2196/19875

**Published:** 2021-04-12

**Authors:** André Mamede, Gera Noordzij, Joran Jongerling, Merlijn Snijders, Astrid Schop-Etman, Semiha Denktas

**Affiliations:** 1 Department of Psychology, Education and Child Studies Erasmus School of Social and Behavioural Sciences Erasmus University Rotterdam Rotterdam Netherlands; 2 Erasmus University College Erasmus School of Social and Behavioural Sciences Erasmus University Rotterdam Rotterdam Netherlands; 3 Department of Public Health, Welfare and Care Municipality of Rotterdam Rotterdam Netherlands

**Keywords:** internet, eHealth, mHealth, mobile phone, lifestyle, obesity, social network, multilevel analysis, physical exercise

## Abstract

**Background:**

Sedentary behavior (SB) and lack of physical activity (PA) have been associated with poorer health outcomes and are increasingly prevalent in individuals working in sedentary occupations such as office jobs. Gamification and nudges have attracted attention as promising strategies to promote changes in health behavior. However, most effectiveness studies thus far lacked active controls, and few studies have tested interventions combining these strategies.

**Objective:**

This study investigates the effectiveness of combining a gamified digital app with physical nudges to increase PA and reduce SB in Dutch office workers.

**Methods:**

Employees in the municipality of Rotterdam (N=298) from two office locations were randomized at the location level to either a 10-week intervention, combining a 5-week gamification phase encompassing a gamified digital app with social support features and a 5-week physical nudges phase, or to an active control (ie, basic digital app with self-monitoring and goal setting). The primary outcome was the daily step count, objectively measured via accelerometers. Secondary outcomes were self-reported PA and SB measured at baseline and at 5, 10, and 14 weeks. Mixed effects models were used to analyze the effects of the intervention on the outcome measures.

**Results:**

A total of 78.5% (234/298) of participants completed the study and provided accelerometer data, whereas 36.9% (110/298) participants completed the self-report measures at 14 weeks. In the gamification phase, step count data were missing for 13.5% (473/3492) of observations in the control and 11.4% (445/3888) in the intervention condition; however, these percentages increased to 39.6% (1154/2910) and 59.6% (1932/3492) at follow-up, respectively. During the gamification phase, intervention participants increased their number of daily steps by 634 (95% CI 154.2-1113.8; *P*=.01) more than participants in the control group, after controlling for relevant factors. Improvements were not sustained during the physical nudges phase (*P*=.76) or follow-up (*P*=.88).

**Conclusions:**

A digital intervention with gamification and social support features significantly increased the step count of office workers compared with an active control. Physical nudges in the workplace were insufficient to promote the maintenance of behavioral changes achieved in the gamification phase. Future research should explore the long-term effectiveness of similar gamified digital interventions.

**Trial Registration:**

International Standard Randomized Controlled Trial Number (ISRCTN) 49129401; https://www.isrctn.com/ISRCTN14881571

## Introduction

### Background

Ample evidence has demonstrated that moderate-to-vigorous physical activity (PA) is associated with improved health outcomes [1]. However, increasing evidence has demonstrated that light forms of PA are associated with decreased risks of cardiovascular disease and all-cause mortality, even after adjusting for levels of moderate-to-vigorous PA [2]. A systematic review has shown that light PA, such as walking (ie, objectively measured step count), is associated with lower risk of obesity and diabetes type 2 [3], and studies have found associations between light PA and lower levels of depression [4,5], stress, and burnout [6]. Moreover, sedentary behavior (SB) has been linked to higher risks of all-cause mortality [7] and reduced life expectancy [7]. SB can be defined as any waking behavior with an energy expenditure ≤1.5 metabolic equivalent of task while in a sitting or reclining posture [8]. SB can be reduced by frequently interrupting the sitting time with light PA, such as walking breaks, which have been shown to reduce the health risks related to SB [9]. Thus, even for individuals meeting guidelines for moderate-to-vigorous PA, regular engagement in light PA is recommended to further reduce all-cause mortality and improve mental and physical health.

Despite these findings, a sedentary lifestyle is an escalating epidemic. Most common occupations have become increasingly sedentary because of technological advancements, and particularly for office workers, workplace sitting patterns are largely responsible for decreases in light PA and increases in SB [10,11]. One study showed that highly educated office workers in the Netherlands spend less time in light PA and more time in SB than workers in other occupations [12], and a recent report found that Dutch workers sit on average for 10 hours per week day [13]. Given that workplace sitting is the largest contributor to decreases in light PA [12] and increases in SB [14], behavior change interventions in this setting can bring considerable benefits at both the individual and societal levels, for instance, through the prevention of health care costs associated with noncommunicable diseases [15].

### Influencing Health Behaviors: Behavior Change Theory and Nudging

Noncompliance with health behaviors can be largely attributed to lack of motivation, insufficient capacity to self-regulate toward one’s goals [16-18], or environmental and policy factors that may limit opportunities for healthy behavior [19-21]. According to the Social Cognitive Theory by Bandura [22], several factors influence motivation for health behavior change, such as whether people are confident in their capacity to change (ie, self-efficacy), which depends on goal characteristics (eg, difficulty), or whether people have social support to change. Social support may involve modeling by family and friends; feedback and support from peers; or social incentives enhancing accountability, competition, and cooperation. People’s capacity to self-regulate can also be influenced by social support and self-efficacy and their ability to self-monitor and their use of planning strategies and reminders [17,18,23]. Socioecological models emphasize the importance of environmental and policy factors for PA, such as walkability and esthetics of the environment, and social norms. Several behavioral change techniques (BCTs) can be used to influence motivational, self-regulatory, or environmental factors to promote behavior change.

### Nudging in the Environment to Promote Motivation and Self-Regulation for Light PA

Certain BCTs attempt to motivate individuals to change by providing information on the risks of their current behavior or on the future benefits of behavior change. However, although these strategies can influence people’s self-reported intention to change, they have, at best, a modest effect on behavior change [24]. Recently, increased attention has been paid to using insights from behavior change theory to help people make better choices by modifying their physical and social environment [25,26] (ie, *nudging).* Nudges are typically BCTs that exploit behavioral and cognitive tendencies to promote a desired behavior, and various interventions have successfully used nudges to stimulate healthy choices in a workplace setting. Motivational nudges can increase motivation for light PA by conveying information on the benefits of walking through an authority figure (eg, a doctor). Nudges can also enhance motivation for PA by influencing the social environment related to PA; for instance, by describing the social norms regarding that behavior or through role models [27,28]. A systematic review found that motivational sign nudges were effective in promoting stair climbing in various settings, including the workplace [29].

Other nudges can help promote the self-regulation of PA goals. For instance*,* point-of-choice prompts are cues that function by interrupting maladaptive habitual behaviors, such as prolonged sitting, and by highlighting opportunities in the environment to engage in alternative health-enhancing behaviors, such as walking breaks. Point-of-choice prompts have been shown to be effective in promoting stair climbing instead of escalator use [30] and walking, thereby reducing SB in the workplace [31]. Workplace nudges typically involve modifications in the physical environment, ranging from changes in the default positions of desks to reduce sitting to the use of motivational or point-of-choice prompt signs incorporating various BCTs to promote walking. However, although nudges are increasingly popular, partially because of their promising cost-effectiveness [32], the effect sizes of nudging interventions tend to be modest [33], and evidence for their effectiveness is still mixed [34,35]. One way to increase the effectiveness of nudges is innovation in intervention delivery, for instance, by including nudges in interactive digital apps [31,36] or by combining it with physical nudges in the work environment, an approach that has been largely overlooked [37].

### Gamification: Improving Digital Interventions to Promote Health Behavior Change

Given the growing use of technology, digital apps are promising avenues for delivering behavior change interventions. Digital interventions provide an empirically supported, convenient, and potentially more cost-effective alternative for reaching large proportions of the public over long periods [38,39]. However, digital interventions still depend on active user engagement to promote behavior change, which is challenging to maintain. Recently, gamification has emerged as a promising persuasive strategy to increase users’ engagement, motivation, and social interaction in digital behavior change interventions [40]. Gamification is an *umbrella term* that refers to the use of game design elements in a nongaming context [41,42]. Gamified digital intervention can flexibly implement a wide range of BCTs, including nudges, such as educational strategies, social support, social comparison, self-monitoring, goal setting, rewards (eg, badges), and personalized feedback, all of which have been associated with greater behavioral change [23,43,44]. Besides promoting self-regulation and motivation for the initiation and maintenance of PA [38,45], gamification can enhance social support and social comparison through competition, cooperation, and salient visualization of others’ behavior (eg, leaderboards) [45,46]. Two recent studies found that digital interventions with elements such as gamification, social support, and social comparison increased light PA in office workers and promoted adequate engagement and adherence with the digital app [46,47].

Despite the promising potential of gamification, recent reviews of gamified digital interventions have highlighted lack of empirical studies comparing gamified digital interventions with active controls (ie, nongamified digital interventions) [45,48]. Although multiple BCTs and nudges can be flexibly incorporated in gamified digital interventions, the effectiveness of such interventions could still be further enhanced through complementary strategies that engage participants outside of the virtual environment. For instance, physical nudges in the workplace, such as *motivational* and *point-of-choice prompts* sign *nudges*, are easy to implement and could serve as a cost-effective way to improve maintenance of the initial behavior change promoted through gamified digital interventions. However, research is needed to explore whether these types of physical nudges could complement and increase the effectiveness of gamification to promote behavior change.

### This Study: MoveMore

We conducted a cluster randomized controlled trial (RCT) to evaluate the effects of *MoveMore*, a 10-week multicomponent intervention, on the PA and SB of office workers. The MoveMore intervention consisted of a 5-week gamification phase that included a commercially available gamified digital app incorporating several BCTs and nudges, such as social support and social comparison, followed by a physical nudges phase for the last 5 weeks, in which physical *motivational* and *point-of-choice prompt nudges* were introduced in the workplace. Intervention effects were compared with an active control encompassing a basic version of the digital app. We hypothesized that during the gamification phase, participants in the intervention condition would increase their levels of objectively measured light PA (ie, daily step count), compared with the control. Similarly, we hypothesized that during the gamification phase, we would observe increases in self-reported light PA, increases in self-reported moderate-to-vigorous PA, and reductions in SB in participants in the intervention condition compared with the control. We expected that improvements achieved during the gamification phase would be maintained during the physical nudges phase and at a 1-month follow-up.

## Methods

### Study Design

To evaluate the effects of the MoveMore intervention, a 2-arm cluster RCT was conducted at 2 office locations in the municipality of Rotterdam, the second largest city in the Netherlands. Each office location was randomly allocated to either the control or the intervention condition to minimize treatment contamination. The study protocol was approved by the Ethics Review Committee of the Department of Psychology, Education and Child Studies, Erasmus University Rotterdam (application number 18-039). The study was registered in the International Standard Randomized Controlled Trial Number Register (ISRCTN 49129401). This study is conducted and described according to the CONSORT-EHEALTH (Consolidated Standards of Reporting Trials of Electronic and Mobile Health Applications and Online Telehealth) checklist (Multimedia Appendix 1) [49].

### Study Setting and Population

Participants were office workers (N=298) from 2 government workplaces in the city center of Rotterdam (office locations A and B). Office location A consisted of a tall office building, with 44 floors accommodating city management and urban development departments, whereas office B consisted of a wide office building, with 5 floors accommodating social development departments. In both locations, government employees belonged to several different occupational groups, including managers, administrative workers, and blue-collar workers. In this field study, it was only possible to recruit departments located in 2 office buildings in the municipality of Rotterdam. Given the limited number of departments involved in this study, our sample size was limited by the number of employees in these departments who were willing and eligible to participate in the research. Considering that the feasible sample size of this field study was larger than that of multiple other PA intervention trials in office workers with similar methodologies [46,50,51], our feasible sample size was considered sufficiently adequate to investigate the effects of this intervention.

### Eligibility Criteria

Given that several components of the intervention were in Dutch, only individuals fluent in the Dutch language were eligible to participate. Additional eligibility criteria included working in a department that was not involved in another PA-related intervention, access to a smartphone capable of running the required digital app, and provision of written informed consent for participation.

### Recruitment

Potential participants were invited via email and social media and through their department team leaders to participate in a study on PA in office workers. Once approximately 150 office workers from each location responded, the invitation was closed. In total, 125 office workers from location A were included in the intervention arm, and 131 office workers from location B were included in the control arm. Participants were enrolled in October 2018 and were followed until February 2019.

### Procedure

**Figure 1:**
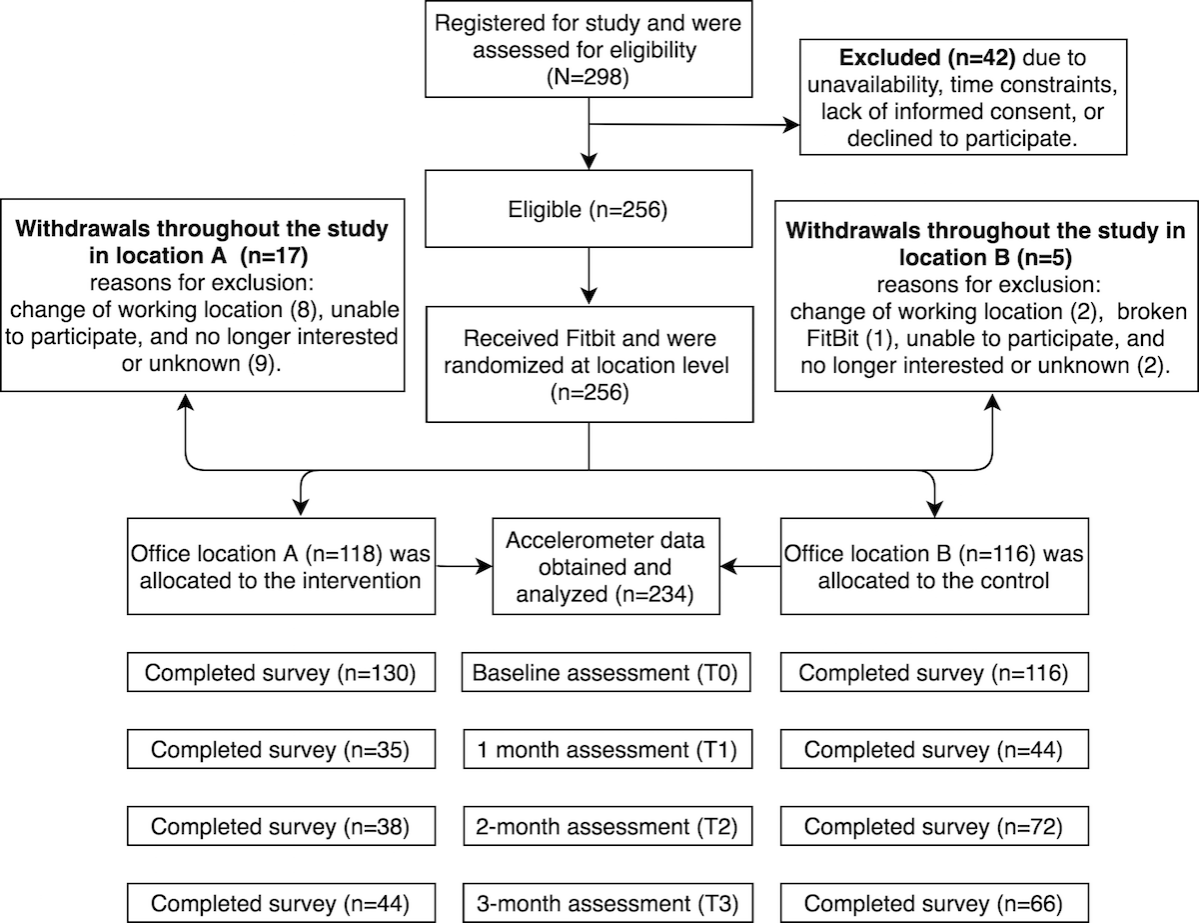
CONSORT (Consolidated Standards of Reporting Trials) diagram displaying the flow of participants throughout the study.

Participants were invited to attend an information session held by a study representative from the municipality, during which potential participants were screened for eligibility criteria, and participants’ baseline measurements and informed consent were collected. Participants received written and verbal explanations of the intervention requirements before providing their consent. Subsequently, participants received a wrist-worn, triaxial accelerometer device (Fitbit Flex) to monitor step count [52] and were shown how to use it in combination with a digital app installed on their mobile phones during the session. The app was available for both iOS and Android operating system and was accessible through a website. Participants authorized their data to be captured for the study. Participants were told that the digital app was intended to support them in becoming more active and that they should use it throughout the day to help them increase their PA. Participants received subsequent questionnaires (Figure 1) via email during the interventions, at 5 weeks after baseline (T1), at 10 weeks after baseline (T2; postintervention), and at 14 weeks after baseline (T3; follow-up). Figure 1 illustrates the flow of participants throughout the study.

#### Intervention and Control

**Figure 2:**
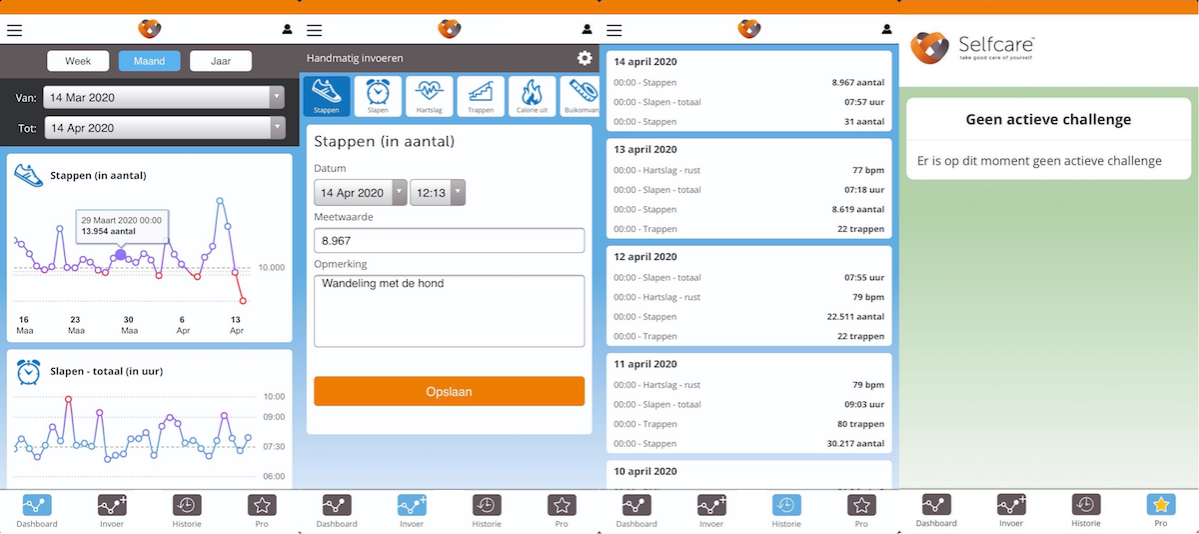
Screenshots of pages available in the basic version of the app used as active control.

**Figure 3:**
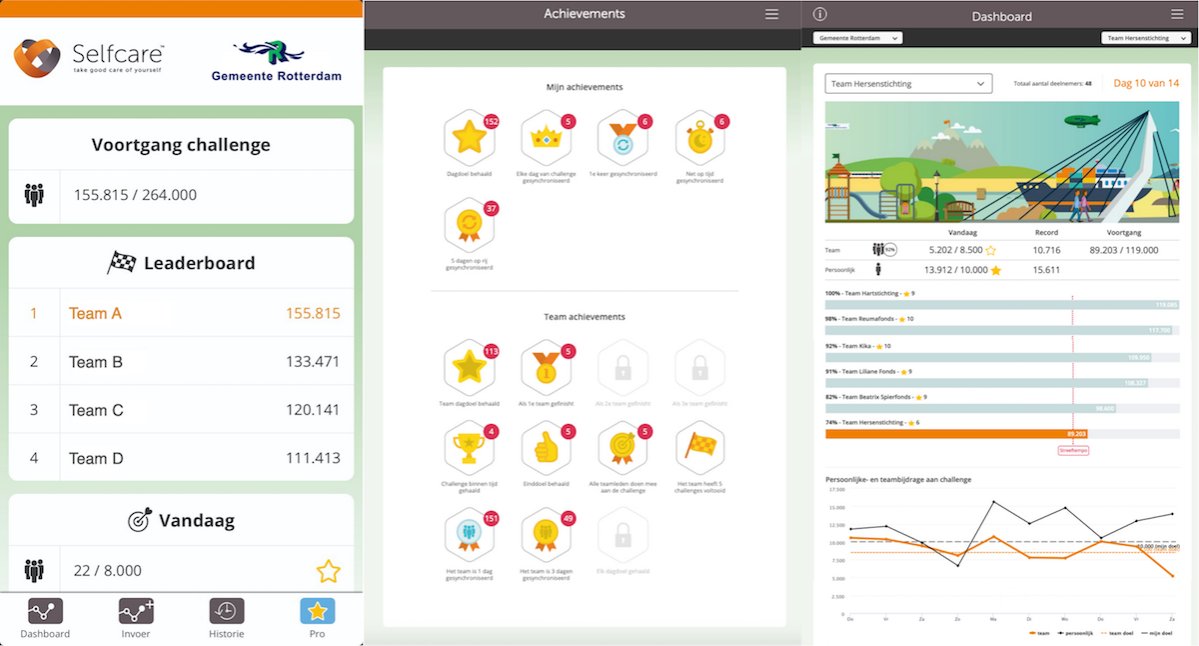
Screenshots of the additional features encompassed in the challenges offered to participants in the MoveMore intervention through the full gamified app.

**Figure 4:**
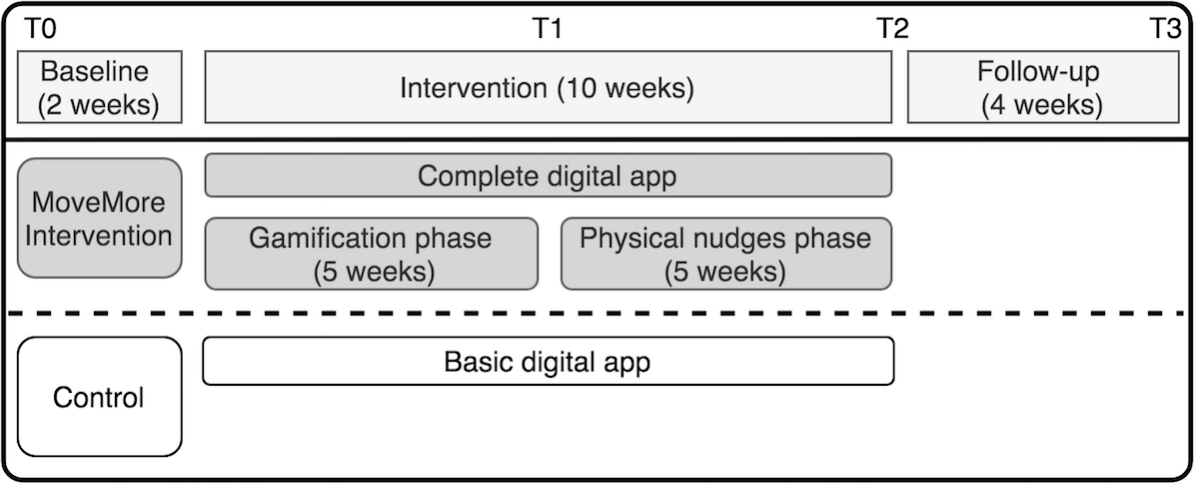
Illustration of study and intervention design. T0 to T3 represent the measuring moments.

Participants in the control location were given a basic version of the app (Figure 2), whereas those in the location receiving the MoveMore intervention were given the full version of the digital app, which included additional features (Figure 3). Both versions of the app were linked to the accelerometer and provided users with a default daily step count goal. Participants were instructed on how to monitor their own daily step count and how to set more challenging daily step goals for themselves (Figure 2). The basic features available in both apps also included weekly personalized feedback to participants via email. In the first 5 weeks of the MoveMore intervention condition (ie, gamification phase), office workers were invited to participate in PA challenges through the digital app, which incorporated elements of gamification and social support and social comparison features. After the gamification phase, physical nudges were introduced to the workplace of participants in the MoveMore intervention for another 5 weeks (ie, physical nudges phase). As one of this study’s aims is to investigate whether physical nudges could promote maintenance of improvements in light PA achieved through the gamification phase, the order of the different study phases was not randomized. An overview of the study and intervention design is shown in Figure 4. The exact BCTs used in the MoveMore intervention and in the active control are described in the subsequent sections and in Table 1 according to the BCT version 1 taxonomy developed by Michie et al [53].

**Table 1:** Behavioral change techniques used in the gamified digital app and the physical nudges (MoveMore intervention) or in the basic digital app (control).

Behavioral components	MoveMore intervention		Control; basic digital app
	Gamified digital app	Physical nudges	
Self-monitoring (ie, accelerometer)	✓^a^	N/A^b^	✓
Information about health consequences	✓	✓	✓
Information about emotional consequences	✓	✓	✓
Self-monitoring	✓	N/A	✓
Goal setting (behavior)	✓	N/A	✓
Discrepancy between current behavior and goal	✓	N/A	✓
Personalized feedback on individual progress	✓	N/A	✓
Personalized feedback on team progress	✓	N/A	N/A
Graded tasks	✓	N/A	N/A
Reward (outcome)	✓	N/A	N/A
Social support	✓	N/A	N/A
Restructuring of the social environment	✓	N/A	N/A
Social comparison	✓	✓	N/A
Prompts and cues	✓	✓	✓
Present information from a credible source in favor of the desired behavior	N/A	✓	N/A

^a^Behavior change techniques applied through the gamified digital app, physical nudges, or basic digital app.

^b^N/A: not applicable.

#### Control Condition

The basic app used in the control condition allowed participants to self-monitor and to set their own daily step goal (Figure 2). The basic app gave participants a default daily step goal of 10,000 steps, which remained the same throughout the study. The basic app also provided participants with weekly personalized feedback on their step count progress via email (Multimedia Appendix 2). This basic app served as an active control because it allowed for an objective assessment of light PA, and its components (ie, self-monitoring, goal setting, and personalized feedback) are effective in promoting PA [54]. The same features provided in the basic apps were available to participants in the MoveMore intervention using the full version of the digital app. The gamified challenges provided to those in the MoveMore intervention were disabled in the basic app provided to those in the control condition (Figure 2).

#### Gamification Phase

In addition to the features included in the basic digital app, the full digital app allowed participants in the MoveMore intervention to participate in team walking challenges (Figure 3). During the first 5 weeks, office workers were invited to participate in 2 challenges, each lasting 2 weeks, with 1 week in between them. To increase participants’ light PA, the challenges incorporated elements of gamification and several BCTs to promote motivation and self-regulation for walking. During the challenge, participants in the MoveMore intervention were allocated to different teams (20-30 subjects), according to the department they worked in. The challenges consisted of a *virtual walking tour* (eg, a roundtrip across Rotterdam) representing a large goal that participants could achieve by attaining their daily goal for 2 weeks. In addition to progressing toward their daily step goals, participants’ daily steps contributed toward their team step goal (ie, set as the number of participants in the team multiplied by their default daily step goal). A leaderboard served to enhance intrateam cooperation and individual accountability while promoting competition between teams (Figure 4). To enhance team identity and motivation, each team was allocated as a representative of a different charity. By earning points and climbing the leaderboard ranks, teams could win gradually bigger prizes for their charity, sponsored by the municipality. The first team earned €100 (US $120), the second team earned €90 (US $108), and so on, with the sixth and last team earning €50 (US $60). These components (ie, teams, leaderboards, and charity representations) were included to restructure the social environment regarding PA, thereby promoting social support and social comparison for walking in the workplace.

In addition to changes in social factors, the game elements of the app were also supposed to motivate participants to walk while increasing their ability to self-regulate their behavior. Similarly, in a game, the challenges started easy and became increasingly more difficult (ie, graded task) to enhance self-efficacy and, therefore, motivation for PA. The default goal for the first challenge was set at 8500 steps, which is easier than the default goal used in the basic version of the app provided to those in the control (ie, 10,000 steps), whereas for the second challenge, participants in the MoveMore intervention were encouraged to reach this more difficult default goal of 10,000 daily steps. Participants could also set more challenging daily step goals. The app rewarded participants with virtual awards for certain individual- (eg, “Daily step goal achieved!”) and team-based achievements (eg, “Your team completed a challenge!”). On the website, participants could access detailed information on their achievements and on their progress with the team challenge, which was illustrated by their virtual avatars crossing the virtual tour scenarios (Figure 3). To promote self-regulation during the challenges, in addition to the weekly feedback on their personal step goals that is provided by the basic digital app, participants in the MoveMore intervention received biweekly newsletters during the challenges via email with updates on the competition and their team’s progress (Multimedia Appendix 2).

#### Physical Nudges

After the gamification phase, physical nudges were introduced in the office workspace of participants in the intervention condition for 5 weeks to promote maintenance of behavior change. These nudges consisted of table signs aiming to (1) further motivate participants to engage in PA and reduce SB and (2) remind participants of the opportunities for PA in their work environment and routine. To achieve the former, motivational nudges incorporating several different behavioral insights were implemented. For example, 2 table sign posters portrayed an interaction between the office physician and an employee and presented the following messages from the physician: “walking breaks are healthy and increase work productivity!” or “My advice: Stand up every half an hour and move a little!” Another type of motivational nudge used social comparison to increase motivation for PA, with the following message: “Half of your colleagues try to move at least 10000 steps per day. What about you?” Motivational nudges were placed in visible locations (eg, on top of tables and eye-level closets) in office spaces and open areas of the intervention location. Complementarily, another type of nudge, namely, point-of-choice prompts, reminded participants of their PA goals, highlighting opportunities for PA in a timely manner and prompting cognitive and behavioral rehearsal. For instance, 2 point-of-choice prompt nudges were placed in the coffee and lunch areas of the workspace with the messages “Grabbing a drink? Perfect moment to be healthy and go for a walking break!” and “Lunch time? Perfect moment to move!” The messages reported have been translated from Dutch (Multimedia Appendix 3). The office workers participating in the intervention were spread across 22 floors. Approximately 5 table sign nudges, including at least one point-of-choice prompt, were placed on every floor in which participants of the intervention worked, for a total of over 110 nudges spread throughout the office building.

### Measures

#### Demographics and Other Variables

The demographic information collected during baseline included participants’ age, gender, weight, length, BMI (ie, calculated from self-reported length and weight), nationality, migrant background (ie, parental nationality), highest educational attainment, occupation in the municipality, weekly number of working days, and working hours.

#### Primary Outcome Measure

The primary outcome measure of walking behavior was the number of daily steps objectively measured using Fitbit Flex accelerometers (objective light PA). Previous studies have determined that the Fitbit Flex accelerometer has acceptable reliability and validity for step count measurements [52].

#### Secondary Outcome Measures

Secondary outcome measures included self-report measures of work time light PA, moderate-to-vigorous PA, and SB. SB at work was assessed using 2-item self-report measures of workplace sitting time and breaks in the sitting time. The self-report measures for assessing the duration of SB (Pearson *r*=0.44, 95% CI 0.24-0.60) and the frequency of breaks from sitting (Spearman *r*=0.26, 95% CI 0.11-0.44) were positively correlated with accelerometer measurements in a sample of desk workers [55]. The item on frequency of breaks, “During a typical work day how many breaks from sitting (such as standing up, or stretching or taking a short walk) during one hour of sitting would you take at work?,” and the item on duration of SB at work, “Please estimate the total time during the last week that you spent sitting down as part of your job while at work or working from home,” were translated into Dutch and assessed for face validity. In our sample, the intraclass correlation coefficients (ICCs) for different measurements of SB duration and SB break frequency during work were 0.44 and 0.60, respectively, indicating poor to moderate test-retest reliability. To assess the intensity and levels of PA in various settings (ie, at work, at home, during active transport), the validated Dutch version of the Short Questionnaire for Assessing Health enhancing physical activity (SQUASH) was used [56,57]. The test-retest reliability of the SQUASH items was poor for assessing hours per week spent in light PA (ICC=0.35) and moderate for items assessing moderate-to-vigorous PA at work (ICC=0.60) and number of days or weeks engaging in at least 30 minutes of moderate-to-vigorous PA (ICC=0.55). Thus, the suboptimal test-retest reliability of some of our self-report measures may have hindered the assessment of intervention effects on secondary outcomes.

### Data Management, Monitoring, and Safety

Except for the baseline, questionnaires were all administered electronically using the web survey platform Qualtrics [58]. Fitbit Flex accelerometer data were obtained through the company responsible for the gamified digital intervention [59] via the Fitbit app and were downloaded at the completion of the follow-up period. Data were exported into R statistical software version 3.5.2 and analyzed using the R package *lmer* [60]. Hardcopy consent forms were stored in locked filing cabinets, and electronic data were stored on password-protected drives accessible by study investigators.

### Data Analysis

In this study, following a period of 2 weeks of baseline measurement, participants’ daily number of steps (ie, primary outcome) was measured continuously for 14 consecutive weeks, resulting in a hierarchical data structure. Daily step count observations (level 1) were nested within participants (level 2), who, in turn, were nested within the departments (level 3). Recent statistical studies simulating variance in longitudinal data have shown that misspecification of the number of levels can lead to biased findings [61]. Therefore, we used a mixed effects model to account for the nested hierarchical structure of the data by including random intercepts for the different levels when the variance at that level was significantly different from zero [62]. As recommended by Haan-Rietdijk et al [61], we used autoregressive models (in combination with Akaike Information Criteria scores) to assess the variance at different levels and determine the levels needed to be included in the model. Given that the variance at the department level was not significant (*χ*^2^_1_=0; *P*=.99), models were only adjusted for the clustering of observations within participants (ie, level 2; *χ*^2^_1_=4934.5; *P*<.001). As we were interested in comparing daily step counts during the different study phases (ie, gamification, physical nudges, and follow-up) with baseline and in investigating potential interactions between intervention conditions and different phases, the study phase was not considered a level but rather included as a predictor in our models.

Considering that multilevel models can handle data missing at random, no missing data imputation was performed, and partially completed records were included in the model to avoid biases associated with a completer-only analysis [63]. In the primary analysis, the first 5 days of data were ignored when estimating the baseline step count to diminish the potential upward bias from estimating higher activity during initial accelerometer use. Observations with less than 1000 steps and more than 60,000 steps were considered missing because evidence indicates that these values are unlikely to represent actual activity [64-66]. Such observations were considered either extreme outliers or the result of forgetting to wear the accelerometer.

To assess the interaction between intervention condition and time and study phases, a repeated measures mixed effects model was employed following the intention-to-treat principles. Models were initially fit with a random intercept for participants, fixed effects of time, study phase (ie, baseline, gamification, nudging, and follow-up), and covariates. When the relationship between our outcome variable and time was quadratic or cubic, quadratic and cubic parameters of time were included in the model as fixed effects. To avoid convergence issues in the primary analysis, time was rescaled to represent 2-week intervals. Covariates initially included in the model were age, sex, parental nationality, work occupation, number of weekly working hours, education, and BMI (ie, calculated from self-reported weight and length). Covariates that were not significant predictors of outcome variables were excluded from the model. Next, the level 2 variable intervention condition (ie, intervention vs control) was included as a fixed effect. Random slopes of the study phase and time per participant were added to the model. When the random slope of the study phase per participant was significant, a 2-way cross-level interaction between the study phase and the intervention condition was included in the final model to investigate the effects of the intervention during each phase. In addition, in an exploratory analysis, we investigated whether intervention effects were influenced by individual differences by examining interactions between intervention effects and relevant covariates. The model used in the primary analysis was refit using secondary outcome measures: (1) the mean number of hours spent in light PA and moderate-to-vigorous PA during work and the number of days engaging in sufficient amount of moderate-to-vigorous PA, as assessed by the SQUASH questionnaire, and (2) 2 self-reported items assessing SB: the average number of sitting breaks taken per hour during work and the mean daily sitting time during work.

## Results

### Demographic Statistics

Table 2 presents baseline descriptive statistics of the study sample per intervention condition. Relative to the control condition, the intervention condition had a significantly higher proportion of male participants and participants of lower educational backgrounds. In addition, participants in the intervention condition weighted significantly more and had significantly higher BMI than participants in the control condition. Participants in the intervention group also logged a lower number of daily steps at baseline, although this difference did not reach statistical significance.

**Table 2:** Sociodemographic and behavioral characteristics of participants at baseline.

Variable			Intervention (n*=*118)		Control (n*=*116)		*P* value
**Behavioral characteristics**							
	Number of daily steps, mean (SD)	10,138 (4643.5)		10,403 (4191.6)		.22	
	Meeting physical activity guidelines (days per week)^a^, mean (SD)	5.1 (2.0)		5.4 (1.0)		.20	
	Hour sitting per week^b^, mean (SD)	30.1 (9.5)		29.2 (10.2)		.45	
	Breaks per hour^b^, mean (SD)	1.8 (1.2)		1.9 (1.3)		.49	
**Demographic characteristics**							
	Age (years), mean (SD)	47.5 (9.6)		45.9 (10.2)		.25	
	Gender (female), n (%)	63 (55.3)		83 (72.8)		*.02* ^c^	
	Weight (kg), mean (SD)	82 (17.8)		75.7 (13.9)		*.003*	
	BMI^d^, mean (SD)	26.9 (5.0)		25.6 (4.5)		*.04*	
	Nationality (Dutch), n (%)	101 (89.4)		104 (92.0)		.57	
	Parental nationality (Dutch), n (%)	88 (77.9)		89 (78.1)		.63	
	Education (higher education), n (%)	78 (69.6)		99 (86.1)		*.005*	
	Work position (highly skilled)^e^, n (%)	105 (97.2)		110 (96.5)		.99	
	Number of weekly working days, mean (SD)	4.4 (0.6)		4.3 (0.5)		.16	

^a^Meeting daily physical activity guidelines was defined as self-reported engagement in at least 30 minutes of moderate-to-vigorous physical activity per day.

^b^The number of hours sitting per week and the number of sitting breaks per hour refer specifically to sedentary behavior during work time.

^c^Italics indicates statistical significance (*P*<.05).

^d^Calculated from self-reported height and weight.

^e^Nonmanual labor occupations, such as managers and administrative positions, were coded as highly skilled.

### Primary Analysis: Daily Step Count

After controlling for relevant covariates and subject-specific differences, our mixed effects model investigated the effects of the intervention condition, time, study phase, and the interaction between study phase and intervention condition on the objectively measured step count of participants. The step count data included in the model were recorded for 109 days from baseline to follow-up. Participants, on average, wore their accelerometers and recorded at least 1000 daily steps for approximately 75 (68.8%; SD 27.8) days throughout the study. During the gamification phase, step data that were missing or had values less than 1000 steps per day represented 13.5% (473/3492) of observations for the control arm and 11.4% (445/3888) for the intervention arm. During the follow-up period, these percentages increased to 39.6% (1154/2910) in the control arm and 59.6% (1932/3240) in the intervention arm, indicating substantial missing data in our sample during the later study phases.

**Figure 5:**
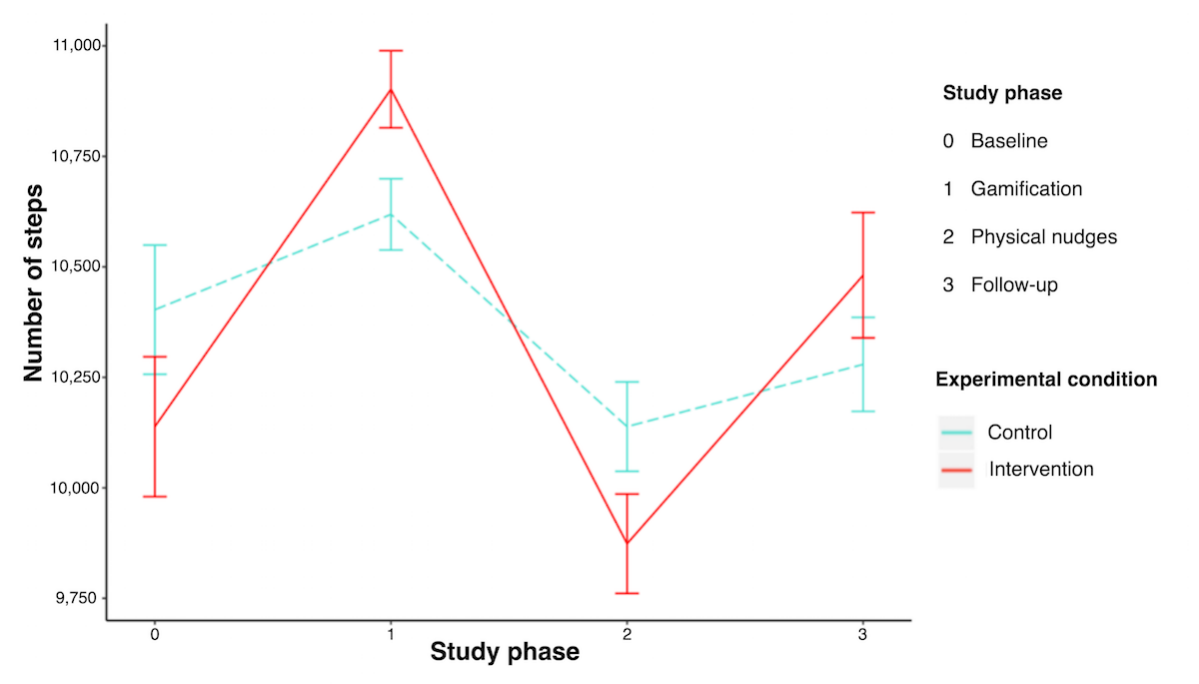
Unadjusted differences in average daily step counts between intervention and control conditions across study phases.

The mean number of daily steps of participants in each condition across the study phases is shown in Table 3 and Figure 5. The first repeated measures mixed model analysis (model 1; Table 3) included the effects of relevant covariates, study phase, time, intervention, and random slopes of time and study phase per participant. Model 1 revealed that the daily number of steps was negatively associated with BMI (*B*=−178.03; SE 38.94; t_168.61_=−4.57; *P*<.001) and positively associated with age (*B=*35.85; SE 18.89; t_172.50_=1.90; *P=*.06) in two-tailed *t* tests. These 2 predictors explained 12.6% of the variance at the participant level (*R*^2^=0.126), with larger BMI and younger age being associated with lower daily step counts overall. The fixed effects of gender, work occupation, number of working hours, education, and nationality (both individual and parental) were removed from the model, as they were not significantly related to step count.

**Table 3:** Means of primary and secondary outcome variables across study phases.

Variable			Baseline (intervention: n=130; control: n=116)		Gamification (intervention: n=35; control: n=44)		Physical nudges (intervention: n=38; control: n=72)		Follow-up (intervention: n=44; control: n=66)
**Intervention location, mean (SD)**									
	Number of daily steps^a^	10,138.3 (4643.5)		10,901.8 (5068.3)		9873.5 (5020.8)		10,481.1 (5035.9)	
	Meeting PA^b^ guidelines^c^	5.2 (2.0)		5.8 (1.6)		5.8 (1.4)		5.6 (1.7)	
	Hours in light PA	26.1 (13.9)		28.4 (12.2)		22.7 (14.4)		20.7 (14.3)	
	Hours in moderate-to-vigorous PA	0.35 (2.2)		0.05 (0.23)		0.08 (0.41)		0.71 (2.24)	
	Hours sitting per week^d^	30.9 (10.7)		32.6 (10.6)		30.4 (9.8)		30.0 (6.8)	
	Number of breaks per hour^d^	1.7 (1.2)		1.5 (0.8)		1.6 (0.9)		1.4 (0.7)	
**Control location, mean (SD)**									
	Number of daily steps^a^	10,403.0 (4191.6)		10,618.6 (4377.3)		10,138.5 (4820.7)		10,279.3 (4387.8)	
	Meeting PA guidelines (days per week)^c^	5.4 (1.9)		5.7 (1.6)		5.8 (1.6)		5.7 (1.7)	
	Hours in light PA	26.4 (13.1)		23.2 (14.1)		23.1 (14.3)		24.7 (13.25)	
	Hours in moderate-to-vigorous PA	0.16 (0.75)		0.22 (0.71)		0.17 (0.81)		0.73 (2.66)	
	Hours sitting per week^d^	29.1 (10.2)		28.9 (8.2)		31.6 (10.7)		29.9 (10.7)	
	Number of breaks per hour^d^	2.0 (1.3)		1.6 (1.0)		1.6 (0.8)		1.6 (0.8)	

^a^The sample size shown refers to questionnaire assessments and does not apply to the number of daily steps measured using accelerometers. In total, 234 participants completed the accelerometer measurements, and on average, the accelerometer was worn on 68.8% of the days.

^b^PA: physical activity.

^c^Meeting daily physical activity guidelines was defined as self-reported engagement in at least 30 minutes of moderate-to-vigorous physical activity per day.

^d^The number of hours sitting per week and number of sitting breaks per hour refers specifically to sedentary behavior during work time.

Given the initial novelty of the gamification and physical nudges phase, which potentially wears off toward the end of each phase, it is plausible that daily step counts oscillated significantly within each study phase. In support of this interpretation, our analysis revealed that linear, quadratic, and cubic effects of time were significant, suggesting that changes in step count within a study phase oscillated across time. Within-subject variability was partially modeled by the fixed effects of time and study phase, whereas the remaining unexplained variance was accounted for by the level 1 error term ε_ij_. Model 1 revealed significant effects of the study phases on step count; however, the main effect of the intervention condition was not significant (model 1; Table 4). These effects of study phases reflect that, after controlling for the effects of time and other covariates, participants from both groups increased their number of daily steps during the novel gamification phase and decreased their number of daily steps during the nudging and follow-up phases, compared with baseline.

**Table 4:** Results of models with and without cross-level interactions predicting number of daily steps.

Parameter	Model 1^a^			Model 2^b^	
	Estimate (SE)	*P* value	Estimate (SE)		*P* value
Intercept	14,003.0 (1233.7)	N/A^c^	14,063.9 (1240.4)		N/A
BMI^d^	−178.0 (38.9)	*<.001* ^e^	−177.1 (39.02)		*<.001*
Age	35.9 (18.9)	.06	36.0 (18.92)		.06
Time^f^	−1261.9 (308.0)	*<.001*	−1259.60 (307.95)		*<.001*
Time^g^	332.5 (81.4)	*<.001*	332.4 (81.36)		*<.001*
Time^h^	−21.2 (6.1)	*<.001*	−21.2 (6.08)		*<.001*
Gamification	856.9 (211.4)	*<.001*	541.9 (241.7)		*.03*
Nudging	−690.9 (326.6)	*.04*	−751.5 (359.0)		*.04*
Follow-up	−1475.3 (406.7)	*<.001*	−1520.2 (441.2)		*<.001*
Intervention	−16.0 (374.8)	.97	−222.3 (416.4)		.59
Intervention × gamification	N/A	N/A	634.0 (244.8)		*.005*
Intervention × nudging	N/A	N/A	98.2 (325.5)		.76
Intervention × follow-up	N/A	N/A	53.49 (381.7)		.89

^a^Model 1: γ_ij_=β0_j_ + β1BMI_j_ + β2Age_j_ + β3Time_ij_ + β4Time_ij_^2^ + β5Time_ij_^3^ + β6Gamification_ij_ + β7Nudging_ij_ + β8Follow-up_ij_ + β9Intervention_j_ + ε_ij_+ μ_ij_. Model 1 (Akaike Information Criteria: 269682) refers to the model without cross-level interactions between the intervention and study phases.

^b^Model 2: γ_ij_=β0_j_ + β1BMI_j_ + β2Age_j_ + β3Time_ij_ + β4Time_ij_^2^ + β5Time_ij_^3^ + β6Gamification_ij_ + β7Nudging_ij_ + β8Follow-up_ij_ + β9Intervention_j_ + β10Intervention_j_Gamification_ij_ + β11Intervention_j_Nudging_ij_ + β12Intervention_j_Follow-up_ij_ + ε_ij_ + μ_0j_+ μ_1j_ Gamification_ij_ + μ_2j_ Nudging_ij_ + μ_3j_ Follow-up_ij_. Model 2 (Akaike Information Criteria: 269679) refers to the model with cross-level interactions between the intervention and study phases. Model 2 significantly improved the model fit (*χ^2^_3_*=8.6; *P*=.04).

^c^N/A: not applicable.

^d^Calculated from self-reported height and weight.

^e^Italics indicates statistical significance (*P*<.05).

^f^Time is rescaled to represent 2-week intervals.

^g^Represents the quadratic function of time.

^h^Represents the cubic function of time.

The changes in daily step count across study phases and time were different between participants, as evidenced by the significant random slopes of the study phase and time per participant detected in model 1. Adding cross-level interactions between the study phase and intervention phase (model 2; Table 4) significantly improved the model fit, indicating that differences between participants in changes in daily step counts across study phases could be explained by intervention effects. Findings from model 2 suggest that differences between participants in changes in daily steps are partially explained by significantly greater increases in daily steps during the gamification phase for participants in the intervention condition than in the control. This was evidenced by a significant interaction between the intervention condition and gamification phase (*B*=634.00; SE 244.81; t_167.52_=2.59; *P*=.005) in a one-sided test, which explained 20.3% of the variance between participants in changes during the gamification phase (*R*^2^=0.203). There were no differences in changes in daily steps between participants in the intervention and control conditions during the nudging phase (*B=*98.23; SE 325.52; t_163.28_=0.30; *P*=.76) or follow-up (*B*=53.49; SE 381.67; t_143.61_=0.14; *P=*.89) in a two-tailed test. Exploratory analysis showed that differences in changes in daily steps between participants could not be explained by individual differences, such as BMI, education, or gender. In essence, our findings indicate that the gamification phase of our intervention was effective in increasing the daily step count of office workers, compared with an active control. However, improvements were not maintained during the physical nudges or during the follow-up phase.

### Secondary Analysis: Self-Reported PA and SB

The sample size and mean values for the secondary measurements at each assessment point are listed in Table 3. Model 1 used in the primary analysis was refitted using secondary outcome measures. Higher BMI was associated with less time spent in SB (*B*=−0.21; SE 0.07; t_115.66_=−2.96; *P*=.003) in a two-tailed test; however, no association was found between the intervention condition or study phase and the time spent in SB. The two-tailed tests revealed that participants in the intervention condition took fewer breaks from sitting than those in the control (*B*=−0.22; SE 0.11; t_229.12_=−2.00; *P*=.046), and participants in both conditions took fewer breaks during follow-up compared with baseline (*B*=−0.34; SE 0.11; t_363.08_=−3.01; *P*=.003). The two-tailed tests also showed that there was no effect of the intervention on hours spent in light PA in the workplace while controlling for parental nationality and age; however, overall, participants engaged in less light PA at the end of the nudging phase (*B*=−4.39; SE 1.45; t_333.73_=−3.03; *P*=.002) and at follow-up (*B*=−3.47; SE 1.40; t_333.98_=−2.48; *P*=.01), compared with baseline. After controlling for BMI, there was no main effect of the intervention for self-reported engagement (hours and days) in moderate-to-vigorous PA (ie, 150 minutes per week). However, a two-tailed test revealed a significant effect of study phase, with participants engaging in more hours of moderate-to-vigorous PA (*B*=0.55; SE 0.19; t_178.83_=2.82; *P*=.005) and more days with sufficient moderate-to-vigorous PA (*B*=0.33; SE 0.16; t_304.05_=2.14; *P*=.03) during follow-up, compared with baseline. Owing to low response rates for web-based questionnaires, it was not possible to refit model 2 with secondary outcomes to examine interactions between the intervention and study phases.

## Discussion

### Principal Findings

This study tested the effects of MoveMore, a multicomponent intervention designed to promote walking behavior (ie, light PA) and reduce SB in office workers. The MoveMore intervention consisted of an initial 5-week gamification phase encompassing a gamified digital app with social support features, followed by a 5-week physical nudges phase, including motivational and point-of-choice prompt nudges. By offering the gamification and physical nudges components separately, we could gain insights into their independent effects and explore whether physical nudges could promote maintenance of behavior change achieved during the gamification phase.

In line with our main hypothesis, significant increases in daily step counts were observed for participants in the intervention condition during the gamification phase compared with participants in the control group. However, contrary to our expectations, improvements in the daily step count for participants in the MoveMore intervention were not maintained during the physical nudges phase or at follow-up. We also hypothesized that similar improvements in secondary outcomes would be observed during the gamification phase for participants in the intervention. Although questionnaires administered in-person at baseline yielded high response rates, we could not investigate differences between intervention and control in changes of secondary outcome measures because of the low response rate in subsequent assessments via email. Nonetheless, overall, participants reported higher engagement in moderate-to-vigorous PA during work at follow-up compared with baseline. Unexpectedly, participants in both conditions reported engaging in less light PA during the physical nudges and follow-up phases than at baseline. Participants in the MoveMore intervention reported taking fewer breaks from sitting than those in the control, and participants in both groups reported taking fewer breaks during later study phases relative to baseline. However, given the poor reliability of the self-report measures in our sample, the validity and generalizability of these findings are limited. Future studies could address these limitations by using more sophisticated accelerometers that measure SB and by using more frequent and less time-consuming measurements that can be integrated in digital apps, such as ecological momentary assessments.

Nevertheless, our main findings suggest that adding gamification components with social support and social comparison features to a digital intervention seems to be an effective strategy for promoting PA in office workers, as evidenced by a significant interaction detected between the gamification phase and intervention condition. Exploratory analysis revealed that intervention effects were not influenced by individual differences, for example, BMI, education, or gender. The short-term effects of the gamification phase on step count were modest (ie, 763.5 increase in average number of daily steps for the participants in the intervention condition compared with 215.6 increase for those in the control) but comparable with previous RCTs evaluating this type of gamified digital intervention [46,67]. Similarly, these studies observed small but clinically significant effects on PA and/or SB. However, systematic reviews of pedometer-based interventions have found that these interventions typically increase PA by approximately 2000 steps per day [68]. Although the gamification phase of the MoveMore intervention resulted in considerable increases in step count, considering the increases reported in other RCTs assessing similar gamified interventions [46,69], these effects may seem underwhelming.

Several factors may explain the smaller effect sizes reported in this study, such as high daily step counts at baseline. Most similar intervention studies recruited inactive adults moving approximately 7000-7500 steps, which is far below the fairly disputed yet often recommended guideline of 10,000 steps per day [46,69,70]. Consequently, larger increases in step counts (ie, approximately 2000 steps) may have been observed in these samples precisely because low levels of PA at baseline allowed for and motivated participants to achieve greater improvements during the intervention period. Comparably, participants in this study walked on average 10,270 steps per day at baseline. Although increasing one’s daily number of steps beyond the recommended guidelines is still beneficial [71], high rates of functioning at baseline may have hindered motivation and limited how many participants in our sample could improve (ie, ceiling effect). Given this ceiling effect, it is comprehensible that increases in the step count of the highly active participants in our sample are lower and more difficult to maintain than those of interventions with inactive participants. Meta-analysis of PA interventions with healthy adults reported that studies with active adults reported lower effect sizes than those with sedentary adults [72]. Given that our sample was already highly active at baseline, the significant improvements in step count observed in participants in the intervention location during the gamification phase highlight the potential of gamified digital interventions to promote light PA.

In addition to the high rate of functioning at baseline, several other factors may partially explain our findings. For example, the use of an active control. Most studies exploring the effects of gamified digital interventions for PA and SB use either nonintervention controls [73] or self-monitoring controls [46,74]. To our knowledge, only a few studies investigating similar gamified digital interventions have used active controls with self-monitoring, goal setting, and personalized feedback [69]. In this study, implementing an active control allowed us to make stronger causal inferences about the effects of gamification and social support features on PA and SB. However, it is well established that the combination of self-monitoring and goal setting alone leads to the initiation of behavior change [54,71]. A meta-analysis of worksite PA interventions found that studies implementing active controls unsurprisingly reported lower effect sizes than those with no intervention controls [75]; thus, the use of active rather than no intervention control is another possible explanation for the relatively smaller effect sizes detected in this study. Another factor that may have influenced our results was the short duration of the gamification phase (5 weeks). A systematic review suggests that PA interventions with longer durations (ie, >24 weeks) are more likely to promote the maintenance of behavior change [76]. Thus, although behavior change was initiated during the gamification phase, 5 weeks may have been too short to form the habit of walking. Due to operational constraints, it was not possible to offer the gamification phase for longer durations in this study; however, future research should explore the effects of gamified digital interventions for longer durations to help establish habit formation.

We anticipated that physical nudges in the workplace could promote the maintenance of the behavior change achieved during the gamification phase. When designing the social norms nudges and point-of-choice prompts, fellow office workers from the intervention location served as role models, which could act as motivators and goal reinforcement [22,77]. Studies have found that combining motivational and point-of-choice prompt nudges was more effective than a control or either strategy alone in increasing stair climbing behavior [78]. However, in this study, combining motivational nudges based on social norms and authority with point-of-choice prompts for walking behavior resulted neither in increases in step count nor in maintenance of improvements achieved through the gamified digital intervention.

A possible explanation for these results may be the differences between the 2 locations. The effectiveness of certain BCTs, particularly nudges, is largely influenced by the context of implementation [33]. The 2 offices randomly allocated to the intervention and control groups differed considerably in terms of location and design. The control location was a wide building located in the city center, with large floor spaces and easy access to stairs and outside areas. Conversely, the intervention location was a tall building, with less surface area per floor and difficult access to outside areas. Participants in the control location had more space to walk indoors, whereas participants in the intervention location worked on multiple floors and may instead have had more opportunities to climb the stairs. Owing to operational constraints, no nudges for stair use could be implemented. Furthermore, weather conditions worsened (ie, lower temperature and more precipitation) throughout the course of the study, which may have discouraged participants from walking outside, maximizing the influence of the physical differences between the 2 locations. In addition, because of the collaborative nature of the study, the nudges used the colors and logos of the municipality of Rotterdam, which are used in other promotional materials found throughout the offices. This may have hindered the attractiveness of the nudges because they easily blended with other unrelated promotional materials.

The ineffectiveness of the nudges may also stem from the possibility that the physical nudges in the workplace in the form of table signs were not sufficiently engaging and motivating, especially when compared with the gamified digital intervention encompassing several BCTs. These findings, however, add support to the emerging evidence indicating that multicomponent interventions incorporating several nudges and BCTs, such as the gamified digital intervention used, are more effective at changing complex behaviors such as PA than interventions relying on only one or a few BCTs, such as the physical nudges used [23]. Most importantly, our findings suggest that gamification can be a useful complementary tool that can be flexibly incorporated to improve intervention effectiveness.

### Strengths and Limitations

Given that reviews of gamified interventions have called for stronger empirical evaluations isolating the impacts of gamification [45], we consider the presence of an active control one of the strengths of this study. This study is the first to combine a gamified digital intervention with physical nudges to promote behavior change. An advantage of this study’s design was the possibility to gain insight into the effects of gamification and physical nudges on initiation and maintenance of behavior change, respectively, although lack of randomization of the order of these components and order effects were the possible drawbacks. The use of objective measurements (ie, accelerometers) was another strength of this study, as self-reported measures of PA are often biased compared with objective measures, leading to false-positive findings [79]. In addition, the MoveMore intervention was implemented in the actual working environment of a large sample of Dutch office workers rather than in a controlled setting, which adds to the ecological and internal validity of our findings. As teams for the gamified challenges were formed according to office departments, a collective motivation for winning may have played a role. However, research has found that gamification is also effective in fostering motivation and promoting PA in adults when applied in individual settings [80]. Our findings, on the other hand, suggest that combining gamification with changes in the social environment that promote social support and social comparison can effectively increase walking in office workers. As shown in a few previous studies, leveraging existing social structures through gamification by, for instance, allocating departments to different teams and stimulating cooperation and competition seems to be an effective strategy for promoting motivation and self-regulation for PA [46,47,81]. Although we recognize that the effects of gamification and social elements of the intervention could not be disentangled, the primary aim of this study is to design an effective intervention rather than isolating these effects.

Nevertheless, this study had several limitations inherent to field experiments, such as the inability to control for extraneous variables and operational constraints with regard to the physical nudges and the time frame of the study phases. Despite the large sample size, given that only 2 worksites were randomized to intervention or control, the limited number of clusters hindered the effectiveness of randomization and comparability between groups. Although we controlled for baseline differences between groups, differences between locations were possible confounders. However, given that the physical attributes of the control location facilitated PA, whereas the attributes of the intervention location hindered PA, it is highly unlikely that increases in step count of participants in the intervention location during the gamification phase can be attributed to physical differences between locations. Due to financial constraints, we could not opt for accelerometers that objectively measured SB. We relied on self-report measures of SB, which had poor reliability in our sample and have been shown to underestimate SB in adults [82,83], possibly explaining why the hypothesized intervention effects on SB were not observed even though they were supported by accelerometer step count data.

With regard to our statistical analysis, although we justified the quadratic and cubic effects of time in our model, we recognize that these higher-order effects are less stable and may be specific to our sample. In addition, dropout during physical nudges and follow-up phases resulted in a considerable amount of missing data, which hindered the assessment and interpretation of intervention effects during those phases. This dropout could simply be a result of timing, as physical nudges and follow-up phases occurred during the holiday season (ie, from December to February). Another possible explanation is that, as research has suggested, although gamification could be effective in enhancing engagement in the short term, users’ motivations are unlikely to be sustained in the long term [48]. Another likely explanation for the high dropout rate in subsequent phases is that, as participants knew that the challenges were only available for a limited time, the gamification phase created a limited-time interest in the intervention, which then diminished once the gamified challenges ended. Owing to privacy agreements, it was not possible to collect data on user experience, functionality, and engagement with the app, which is a limitation of this study. Future research should investigate the long-term effects of gamified interventions while measuring user experience, engagement, and other possible mediators of intervention effectiveness.

Although our findings support the effectiveness of integrating gamification and social support features in digital interventions to promote light PA in active office workers, further research is needed to confirm the generalizability of our findings in more at-risk populations, such as inactive adults and adolescents from disadvantaged backgrounds. Given the unexpected effects of physical nudges, future studies should carefully consider the design and context of nudges. Nudges have gained attention because they are cost-effective in influencing people’s behavior momentarily. However, further research is needed to explore how physical nudges can be more effectively combined with other interventions to promote the maintenance of complex behaviors, such as PA, in the short and long term. For example, offering gamified digital interventions and physical nudges simultaneously, rather than sequentially, may increase the intervention effectiveness. Research on gamification and nudging is still in its infancy. Future research with gamified digital interventions should continue innovating with the design of digital interventions by, for instance, testing different forms of social support and gamification components. Finally, future research on digital interventions should investigate complementary strategies to promote long-term maintenance of behavioral change, such as increasing engagement by tailoring the interventions to the participants’ needs or using guided approaches in incorporating personal coaches or peer support.

### Conclusions

Compared with an active control consisting of a digital app, including self-monitoring and goal setting, a gamified social support–based digital intervention was effective at promoting light PA (ie, objectively measured number of daily steps) in a sample of active office workers. Given the high prevalence of sedentary lifestyles and the associated health problems, both of which are costly to health care systems, even small improvements in light PA can have considerable effects at the population level. This study was one of the first to compare the effects of gamification with an active control and to test its effects on PA and SB of office workers. Our findings demonstrate that gamification can effectively complement the BCTs (eg, social support and social comparison) and nudges used in digital interventions to promote clinically significant improvements in PA, even beyond the recommended guidelines of 10,000 steps. Although more research is needed to establish its long-term effectiveness, policy makers should explore the use of gamified digital interventions with social support features as a promising strategy to promote behavior change and improve the health of the population. Physical nudges in the workplace were insufficient to promote the maintenance of behavior changes achieved during the gamification phase. Further research should explore how gamified digital interventions can be better leveraged to promote long-term behavior change, for instance, by investigating how to optimally tailor digital interventions to users’ needs.

## Appendix

Multimedia Appendix 1 CONSORT-EHEALTH checklist (V 1.6.1).

Multimedia Appendix 2 Pictures displaying the components included in the basic and gamified versions of the digital apps used.

Multimedia Appendix 3 Pictures displaying the original nudges (in Dutch) introduced in the workplace of participants in the MoveMore intervention condition during the physical nudges phase of the study.

## Abbreviations

BCT: behavioral change technique
CONSORT-EHEALTH: Consolidated Standards of Reporting Trials of Electronic and Mobile Health Applications and Online Telehealth
ICC: intraclass correlation coefficient
PA: physical activity
RCT: randomized controlled trial
SB: sedentary behavior
SQUASH: Short Questionnaire for Assessing Health enhancing physical activity
