# Supplementary material for: Combining Web-Based Gamification and Physical Nudges With an App (MoveMore) to Promote Walking Breaks and Reduce Sedentary Behavior of Office Workers: Field Study
Source: J Med Internet Res. 2021 Apr 12;23(4):e19875. doi: 10.2196/19875 (PMC8076996; doi:10.2196/19875)

## **Multimedia Appendix 2. Pictures displaying the components included in the basic and gamified versions of the digital apps used.**

This document contains the additional figures illustrating the components in both the basic and full gamified version of the digital applications utilized Move More study. Additional Figures 1 and 2 are example of pages included in both the basic version and the full gamified version of the application, hence these pages were available for participants in both the intervention and control condition. Additional Figure 2 shows the personalized feedback provided via email to participants in both the intervention and control. The newsletter and additional figures 3, 4 and 5 were only included in the full gamified version of the application, and were therefore only available for participants in the MoveMore intervention condition.

**Additional Figure 1.** Example of pages displayed in the both the full and the basic application displaying users’ personal step goal and their daily step count.


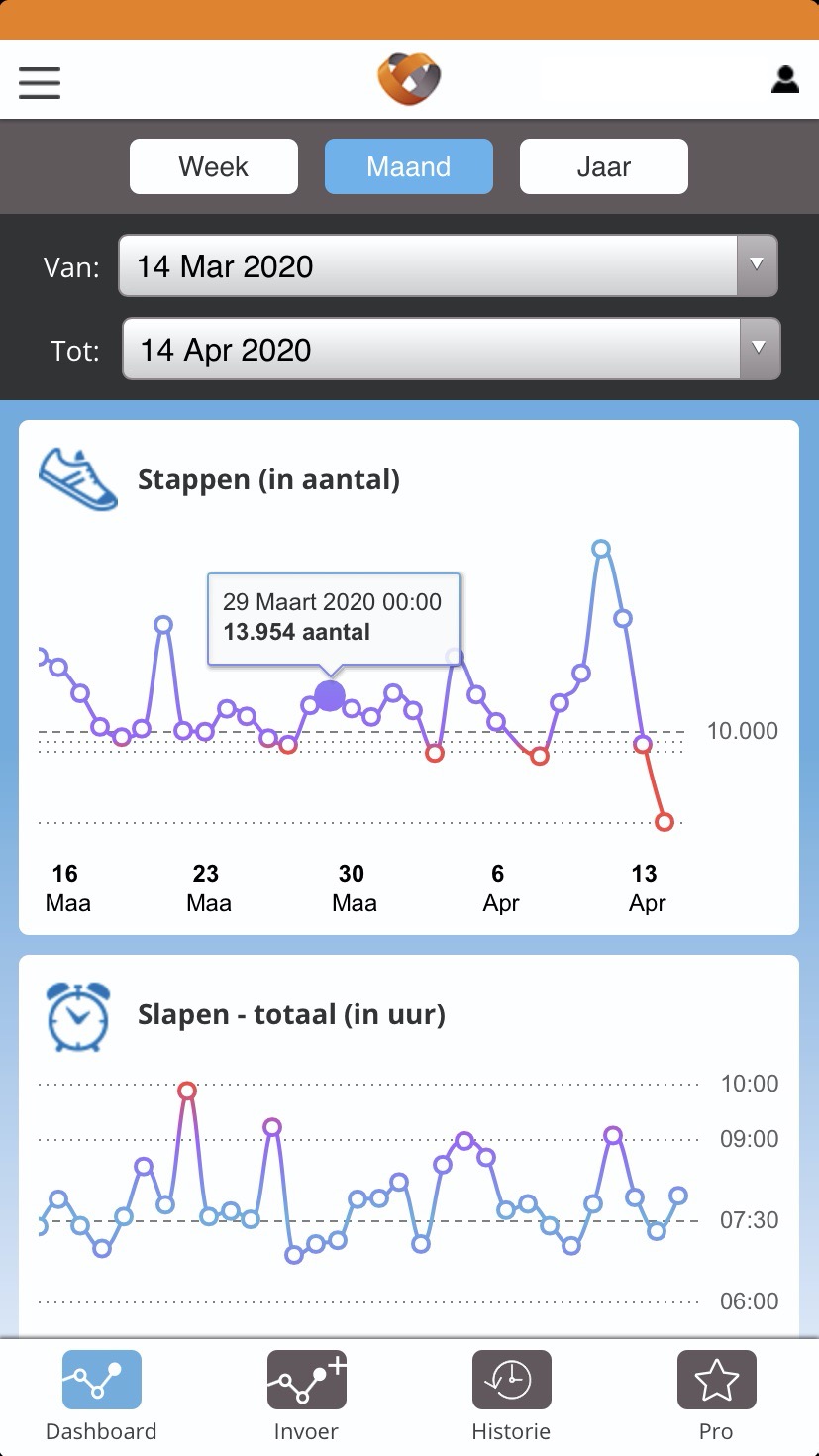

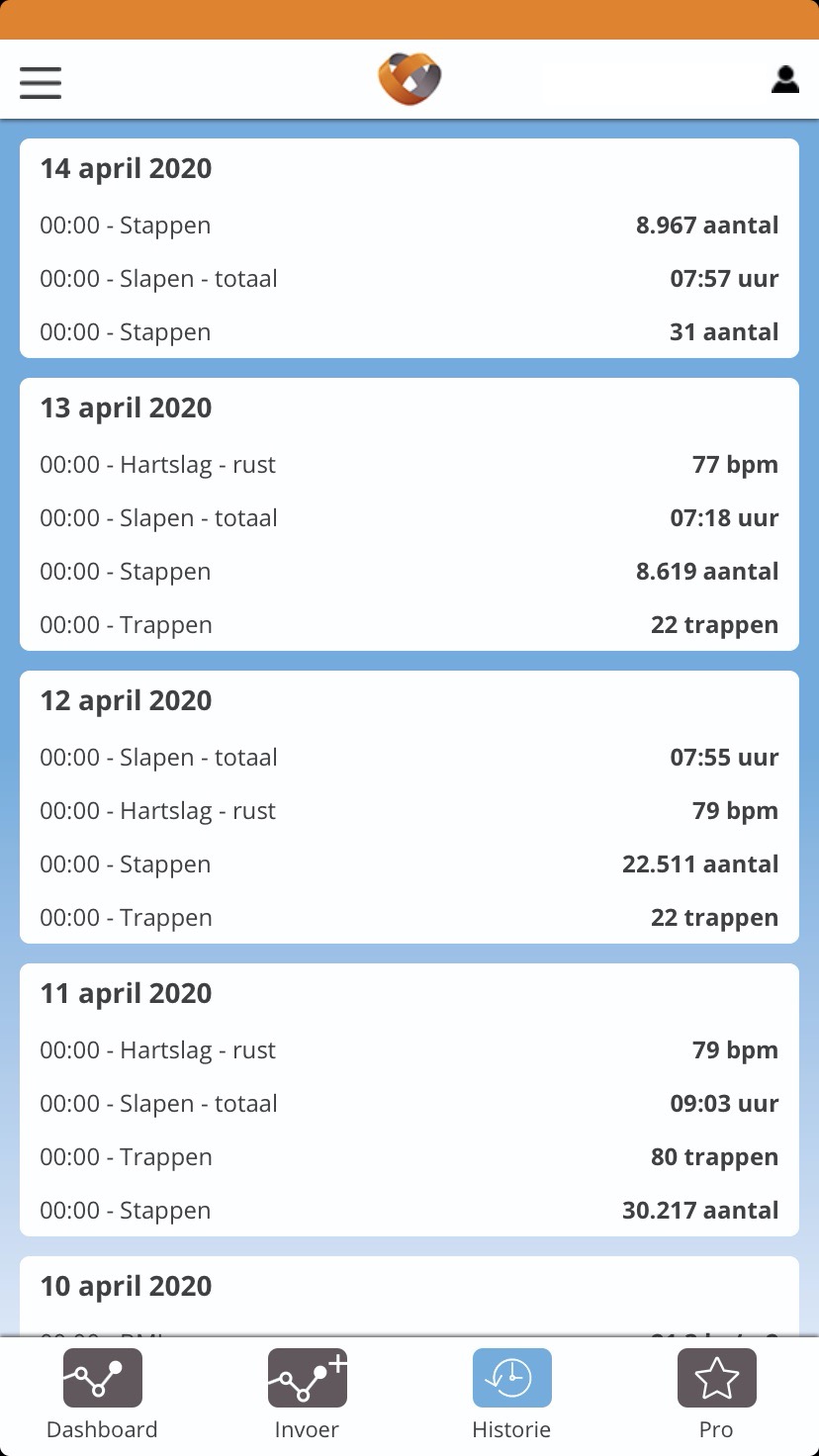


**Additional Figure 2.** Example of pages included in the both the full and the basic application in which users can set their own daily step count goal.


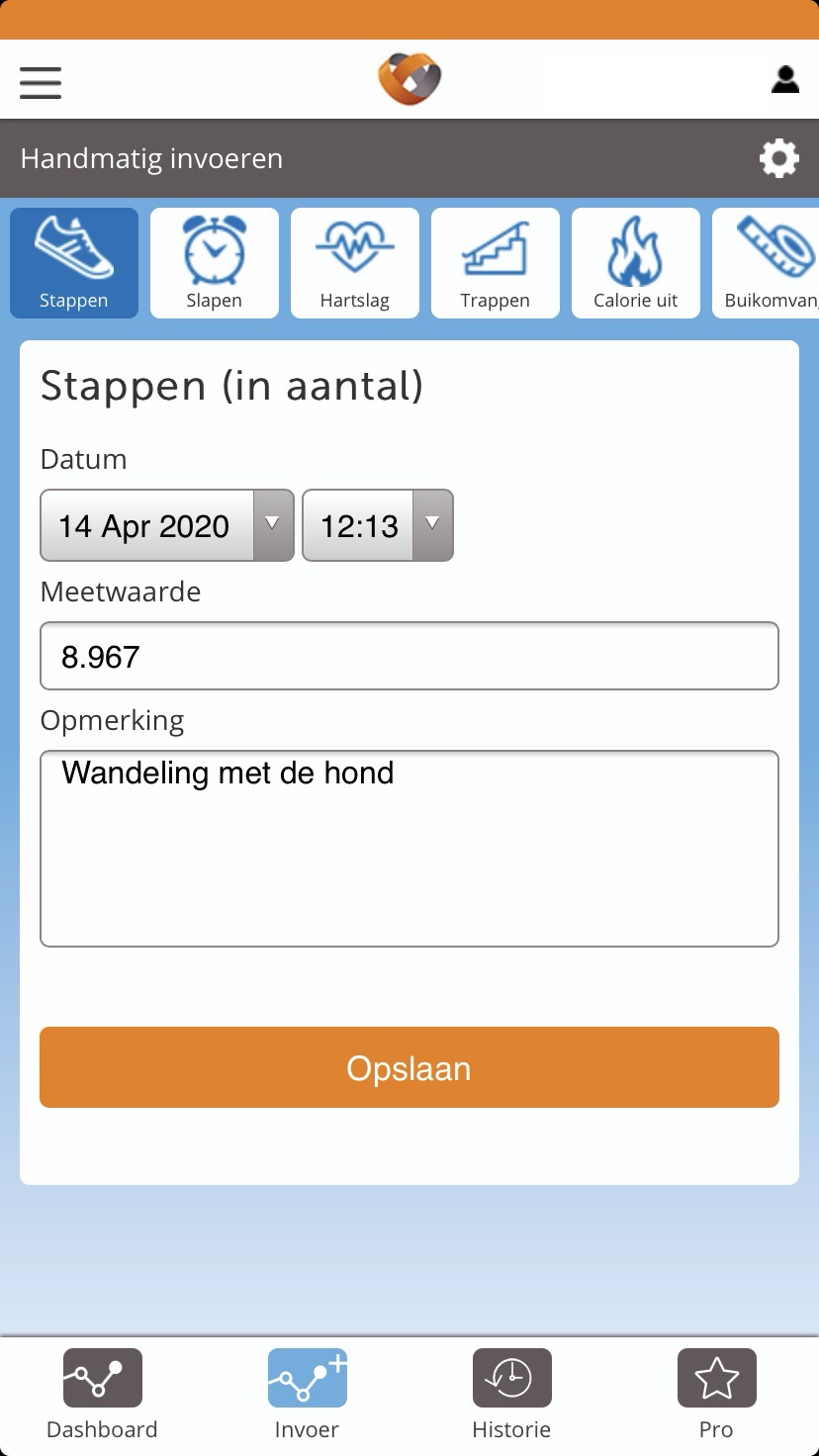


**Additional Figure 3.** Example of personalized feedback on participant’s progress with their step goals sent via email to participants in using both the full and the basic version of the application (i.e. intervention and control conditions).


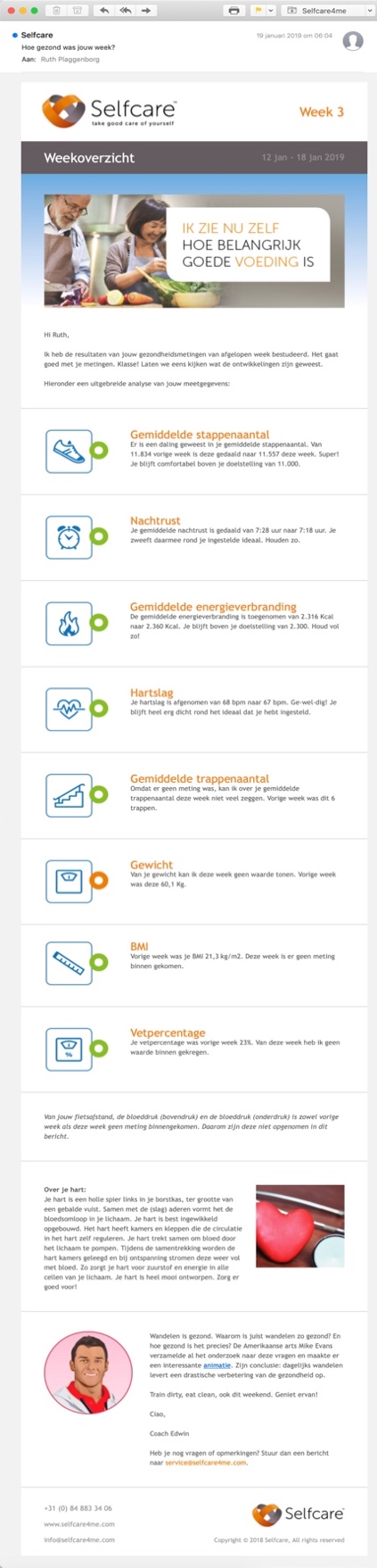


**Additional Figure 4.** Example page included in the full gamified version of the application displaying the user’s team’s step goal and progress. This page also included a leader board in which the user’s team position is compared with other teams.


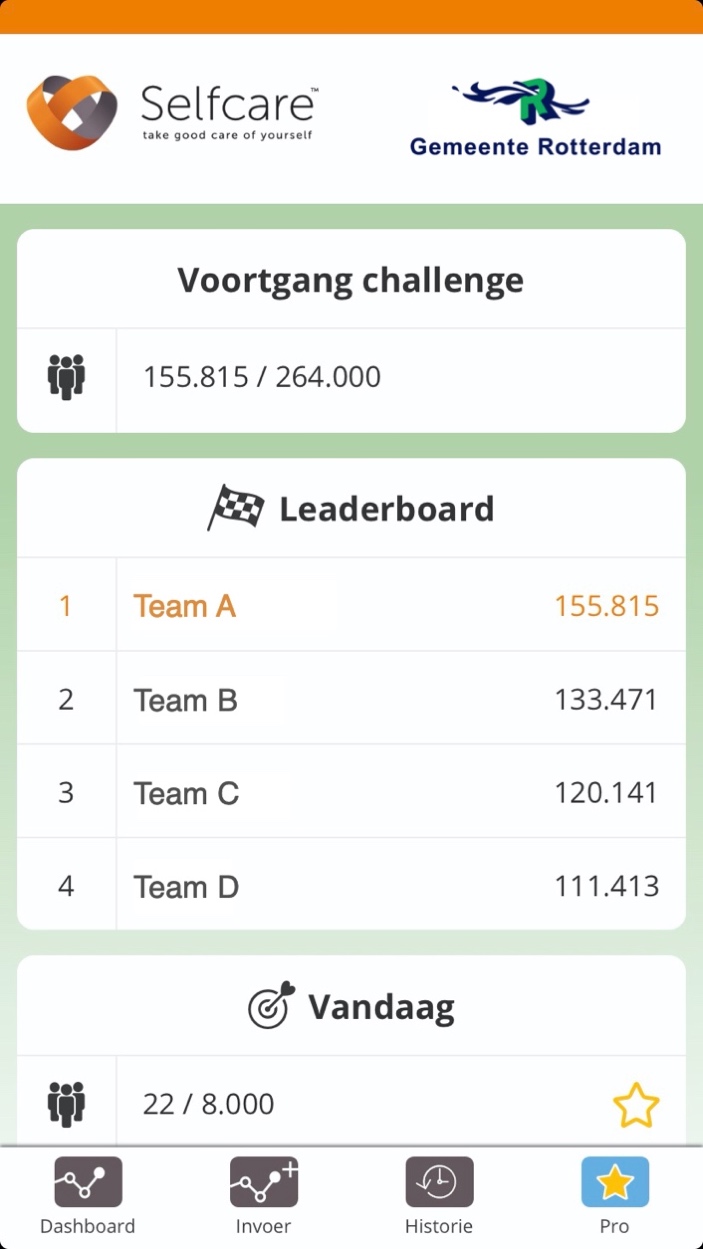


**Additional Figure 5.** Example of page in the full gamified version of the application displaying personal and team achievements (i.e. virtual badges) that the users can earn during the challenges.


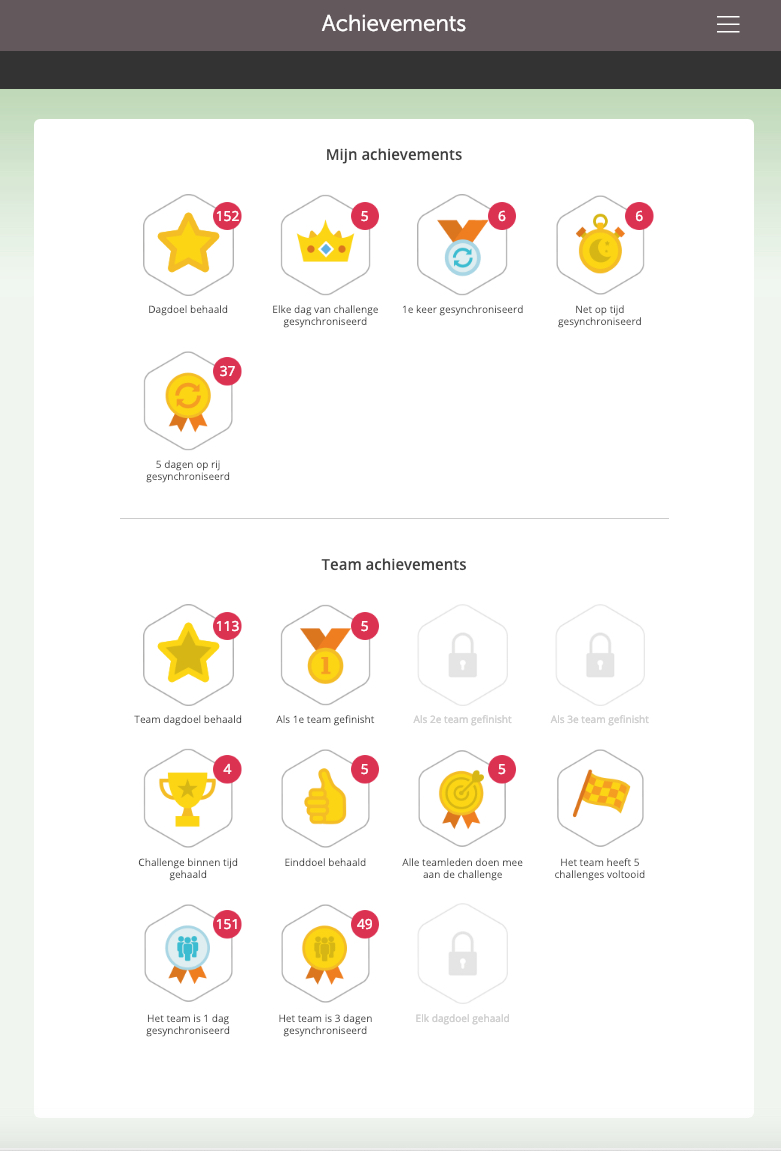


**Additional Figure 6.** Example dashboard page included in the full gamified version of the application summarizing the information available to the user. The page displayed the user’s personal step goals and progress, as well as their team’s step goal and progress. This page also displayed to users all the achievements they and their team had earned, and showed a leader board in which the user’s team position is compared with other teams.


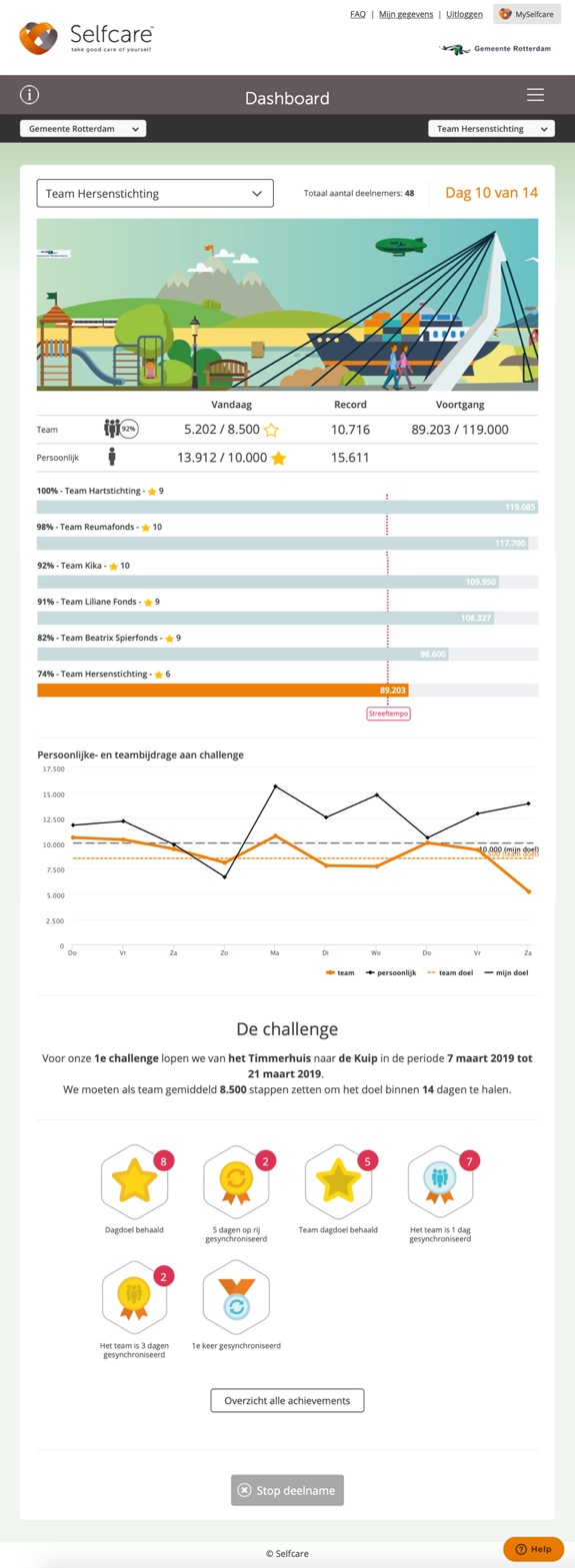


**Additional Material: Newsletter.** This is an example of the newsletter provided to participants in the MoveMore condition via email every two weeks during the gamification phase. The news brief updated participants regarding their progress with the gamified challenge, as well as provided them with several tips on how to improve their use of the digital application. The newsletter also included information to motivate participants to be physically active, and tips on how to move more throughout the day. Finally, this email news brief also included the link to the online survey assessing secondary outcomes.

**
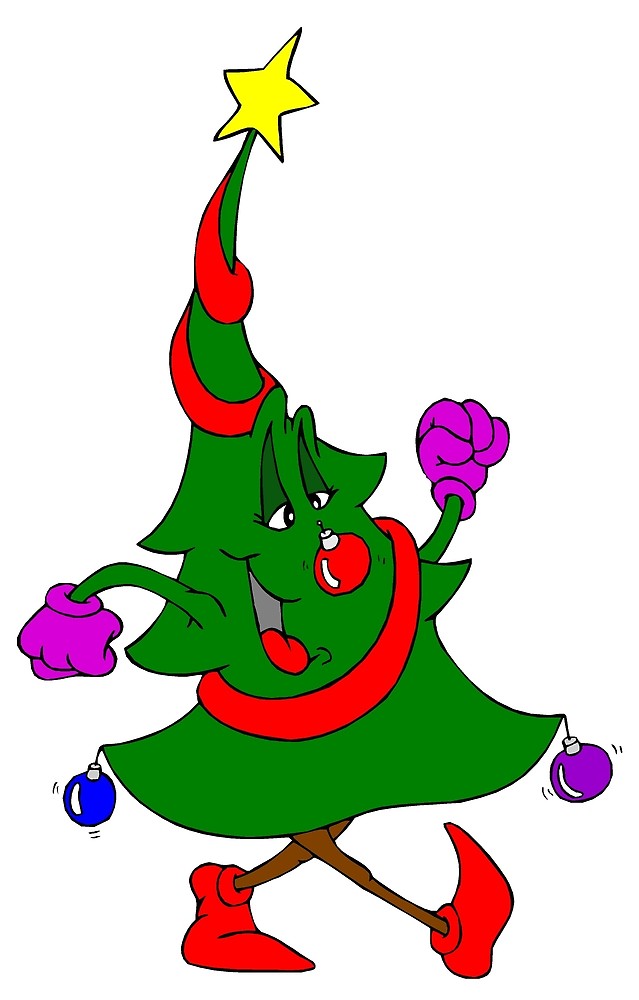
**

**Vrije dagen en bewegen!**

**Kerst staat voor gezellig samenzijn. Dit kan binnen onder de kerstboom, maar ook in de frisse, of beter gezegd verfrissende, buitenlucht. Het kerstdiner zal na het lopen van een wandeling nog lekkerder smaken. Vrolijk kerstfeest!**

De dagen rond de kerst zijn natuurlijk bij uitstek geschikt om wat extra te bewegen! Loop eens een extra rondje door de buurt om de lichtjes te bewonderen of maak één van de door Metro geselecteerde [kerstwandelingen](https://www.metronieuws.nl/nieuws/rotterdam/2015/12/kerstwandelen-in-rotterdam-vijf-tips) in Rotterdam.

**Het onderzoek**

Wij zijn natuurlijk erg benieuwd of het dragen van de Fitbit en hiermee het inzicht in je eigen beweeggedrag er voor zorgt dat je meer gaat bewegen en of je je hierdoor ook vitaler voelt. Bij de Kick off van Move More is uitgelegd wat er met betrekking tot het onderzoek van jullie wordt verwacht.

**Challenge en goede doelen!**

**Team Hersenstichting is in de Bruggenloop met een teamgemiddelde van ruim twaalfduizend stappen per dag als eerste over de finish gekomen! Hiermee hebben ze hun grootste concurrent het Beatrix Spierfonds deze keer verslagen.**

**Alle teams bedankt voor jullie inzet (het totale gemiddelde was wederom boven de 10.000 stappen per dag), dus heel goed gedaan allemaal!**

**Hieronder de verdeling van het prijzengeld voor de goede doelen, we gaan namens jullie allemaal zorgen dat de bedragen in de vorm van VVV bonnen bij de fondsen terechtkomen!** Let op, de pilot en het onderzoek lopen nog door tot en met januari. Dus blijf je fitbit in ieder geval de komende anderhalve maand nog dagelijks dragen en synchroniseren.


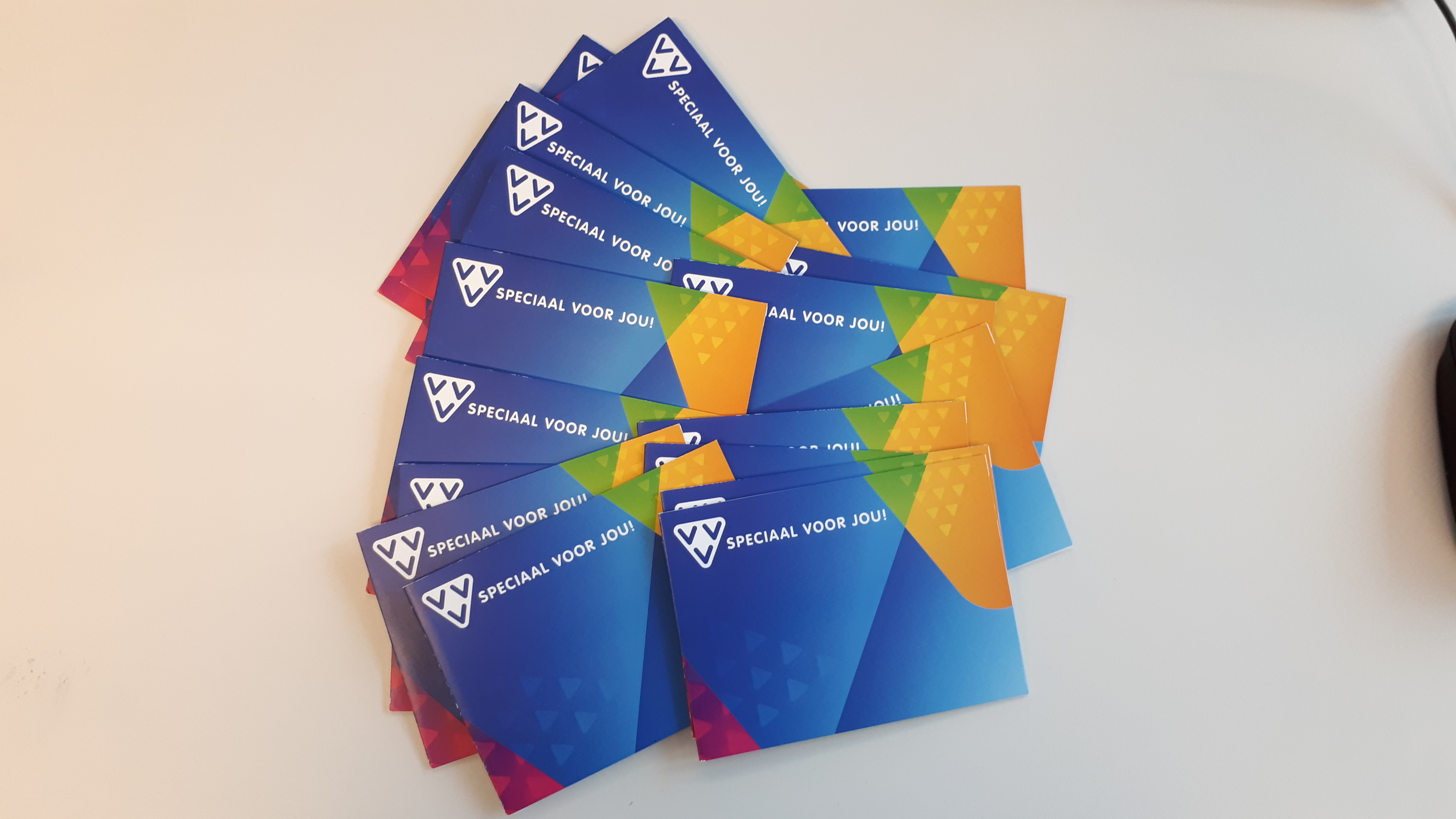


|  | **Beklimming Rotterdam** | **Bruggenloop** | **Totaal prijzengeld** |
| --- | --- | --- | --- |
| Beatrix Spierfonds | **1^e^ prijs €100,-** | **2^e^ prijs €90,-** | **€190,-** |
| Hersenstichting | **2^e^ prijs €90,-** | **1^e^ prijs €100,-** | **€190,-** |
| Liliane Fonds | **3^e^ prijs €80,-** | **4^e^ prijs €60** | **€140,-** |
| Reuma Fonds | **4^e^ prijs €70** | **5^e^ prijs €70,-** | **€140,-** |
| Hartstichting | **5^e^ prijs €60,-** | **3^e^ prijs €80,-** | **€140,-** |
| Kika | **6^e^ prijs €50,-** | **6^e^ prijs €50,-** | **€100,-** |

**Werk je de komende weken gewoon door?**

**Een (bijna) uitgestorven kantoor biedt natuurlijk extra mogelijkheden om een rondje door het gebouw te lopen of eens een andere locatie (en mogelijk tijdens een lunchwandelingetje ook de omgeving hiervan) te verkennen.**

**Stille alarmen instellen, oproepmeldingen en andere functies**

**Wist je dat de fitbit zo kan instellen dat hij zachtjes trilt om je wakker te maken of om je te waarschuwen?**

**Als je op een vlek zit en je je collega’s om je heen niet wil storen met jou gezellige beltoon kun je je telefoon op stil zetten en oproepmeldingen inschakelen. Sowieso is een telefoongesprek natuurlijk hèt moment om de benen even te strekken en naar een rustig plekje te wandelen.**

**Ook kun je herinneringen om te bewegen instellen, kijk voor meer functies in een** [handleiding](https://www.gebruikershandleiding.com/Fitbit-Flex2/preview-handleiding-836903.html)**.**

### Fitbit lelijk?

### Google even naar de mogelijkheden, want voor een paar tientjes is dat probleem opgelost. Misschien dat bol of ali nog kan zorgen dat er een leuke accessoire onder de kerstboom ligt, zodat je mooi kunt blijven bewegen in het nieuwe jaar!

###
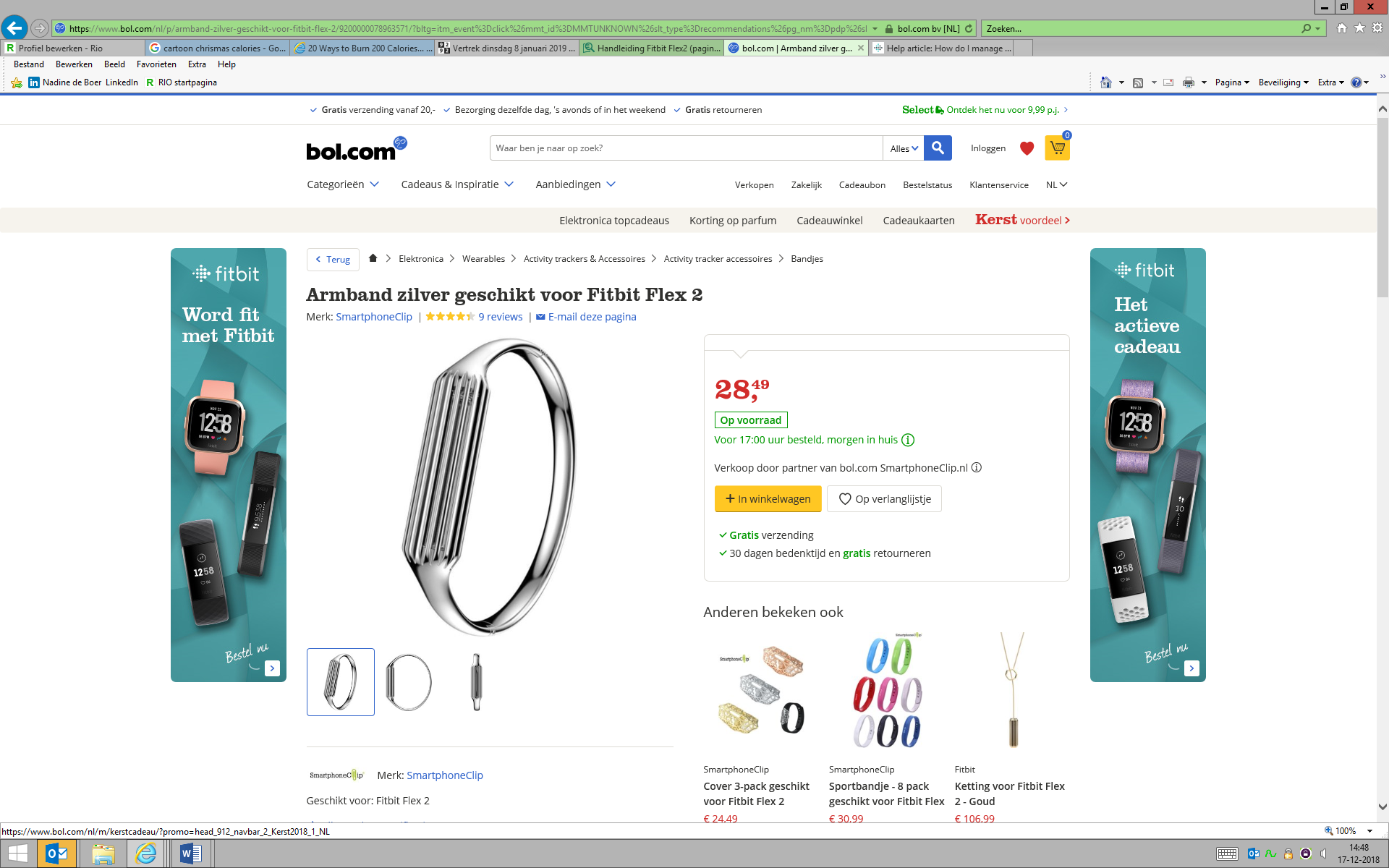

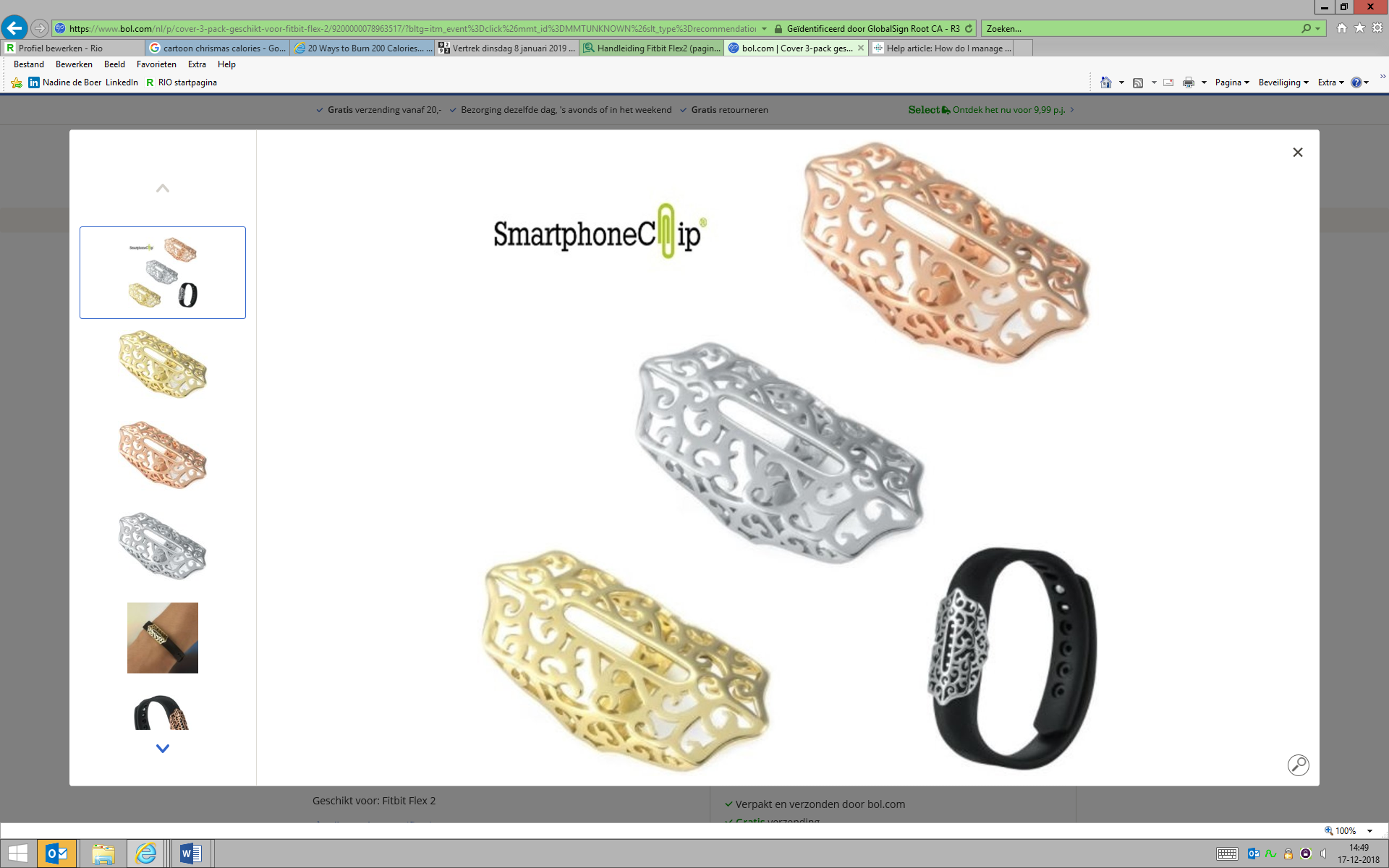

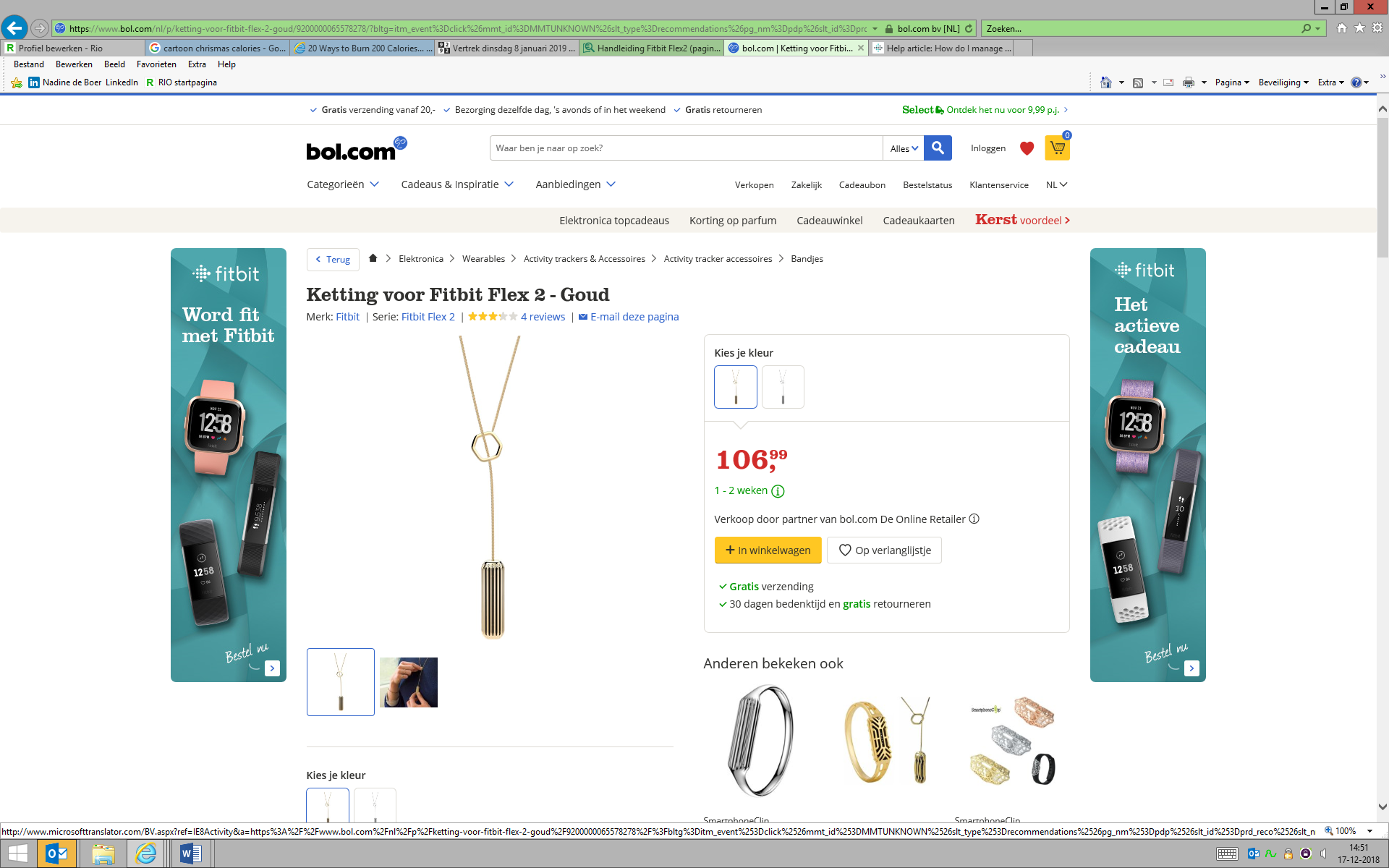


**Vragen of suggesties?**

De Selfcare Helpdesk staat nog steeds voor je klaar. Heb je een vraag? De [Selfcare Helpdesk](http://selfcare4me.us10.list-manage.com/track/click?u=5f81c30e1306a7b9f002625ec&id=f6b7dae669&e=46b7453f91) heeft het antwoord!

Heb je een vraag over het Move More of een suggestie? Mail dan naar [m.snijders2@rotterdam.nl](mailto:m.snijders2@rotterdam.nl).

### Wij wensen jullie namens het Move More Team allemaal

### hele fijne feestdagen en een gezond en beweeglijk 2019!

### Tot in het nieuwe jaar!


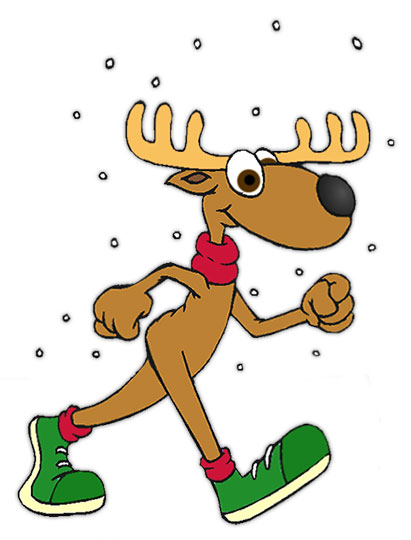

Supplement: Multimedia Appendix 2 [file jmir_v23i4e19875_app2.docx]
