# Supplementary material for: Combining Web-Based Gamification and Physical Nudges With an App (MoveMore) to Promote Walking Breaks and Reduce Sedentary Behavior of Office Workers: Field Study
Source: J Med Internet Res. 2021 Apr 12;23(4):e19875. doi: 10.2196/19875 (PMC8076996; doi:10.2196/19875)

Gemeente Rotterdam

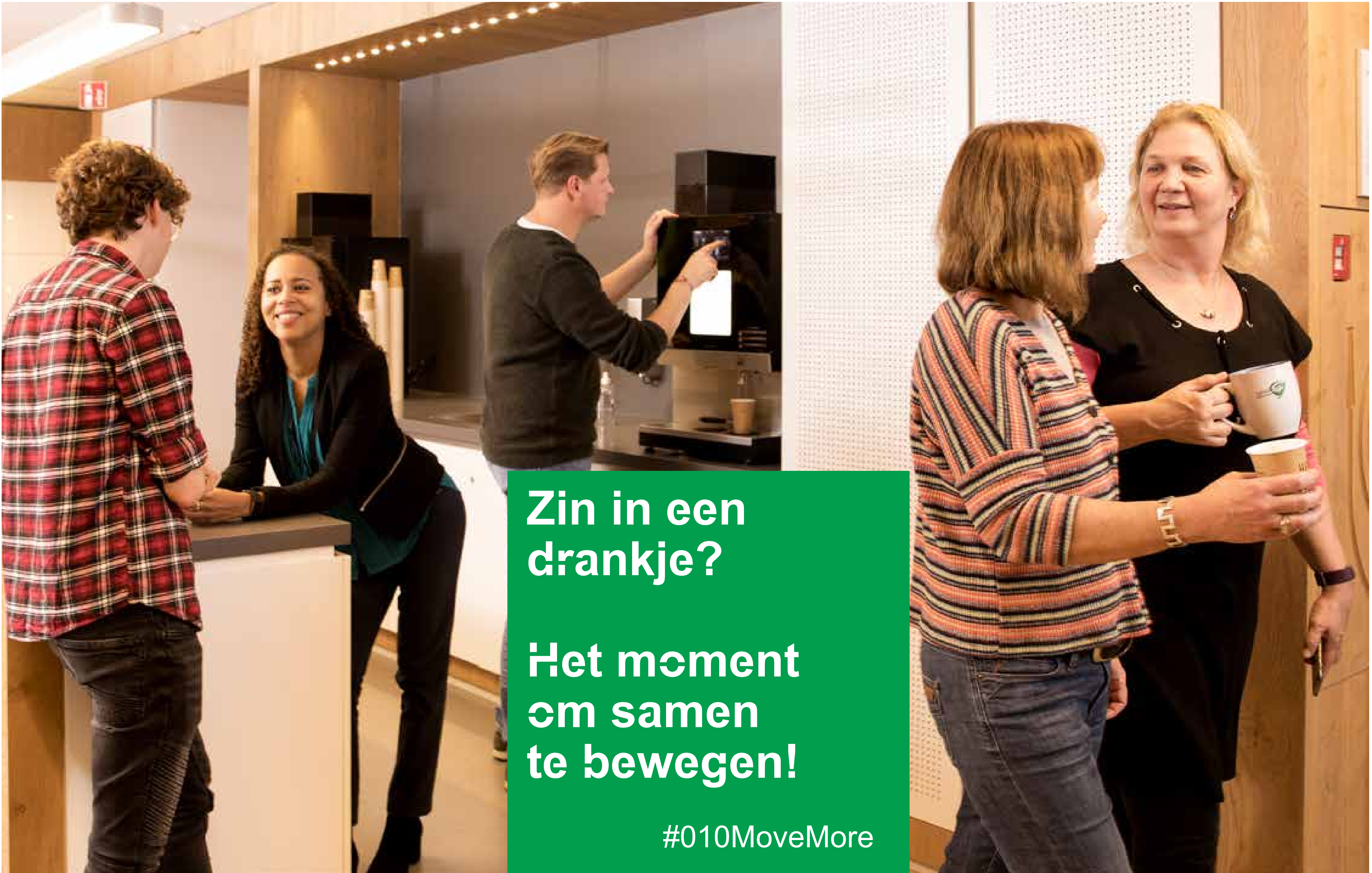

Zin in een drankje?

Het moment om samen te bewegen!

#010MoveMore

Gemeente Rotterdam

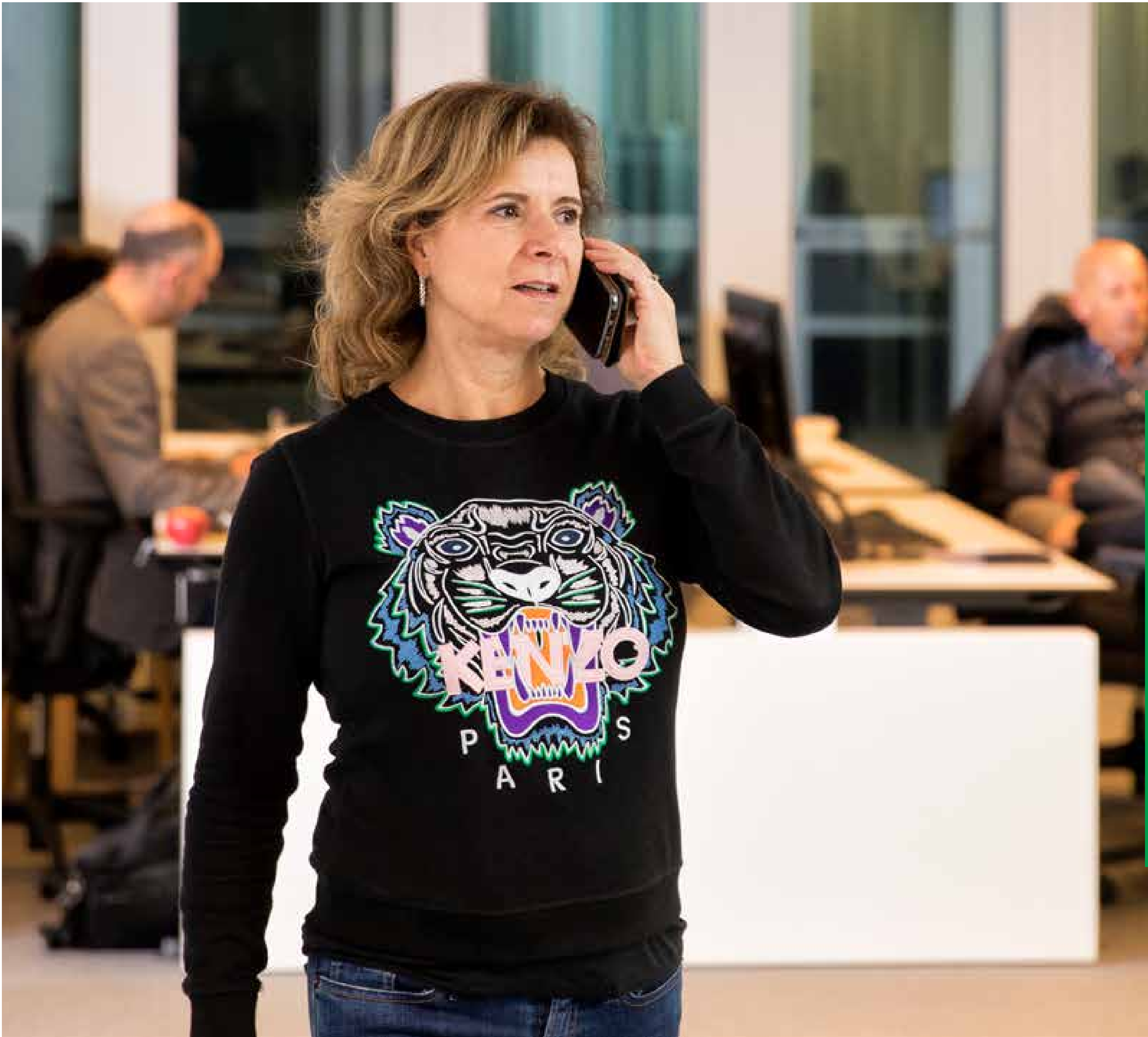

Niet storen?

Loop even een rondje!

#010MoveMore

Gemeente Rotterdam

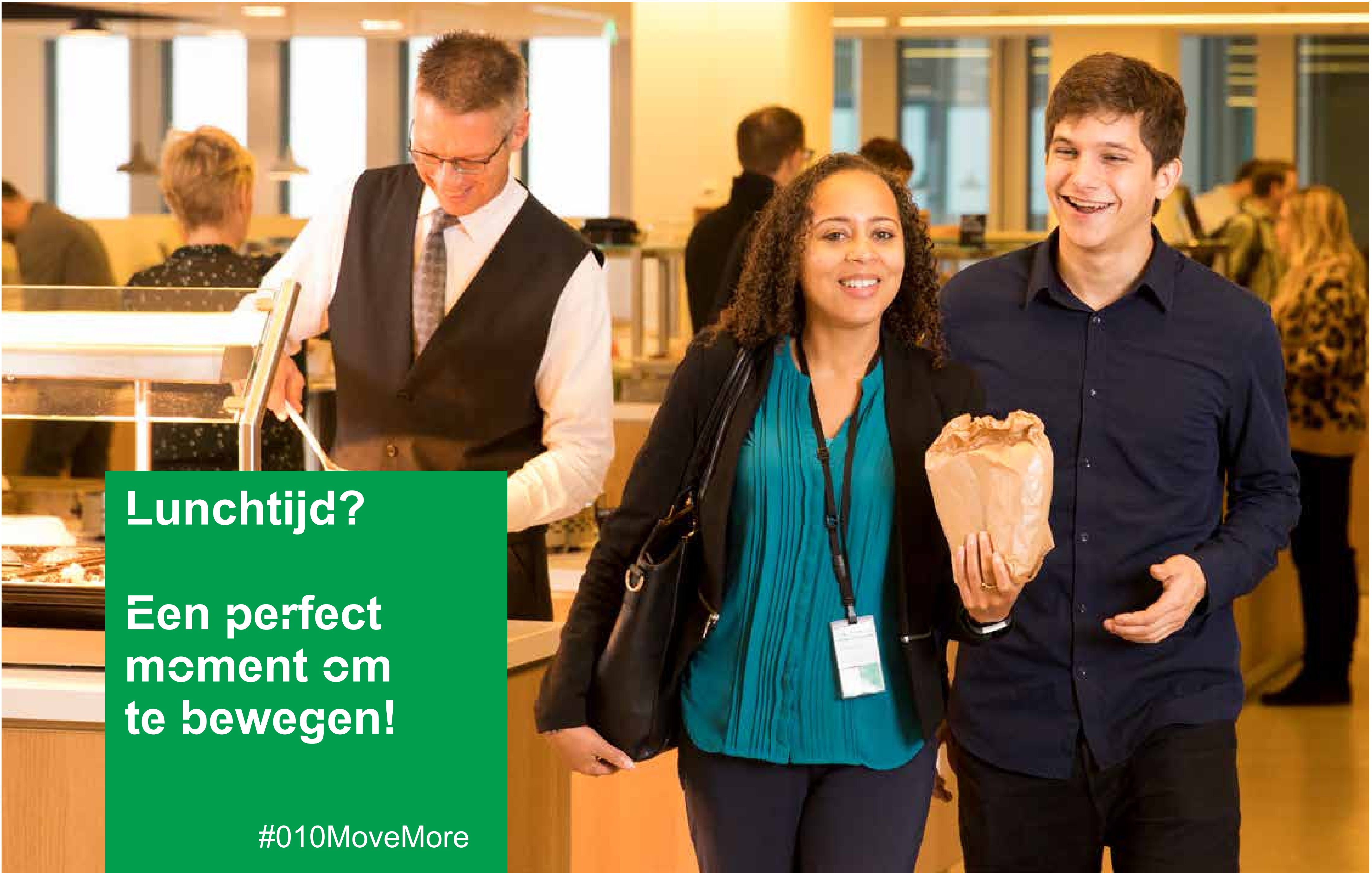

Lunchtijd?

Een perfect moment om te bewegen!

#010MoveMore

Gemeente Rotterdam

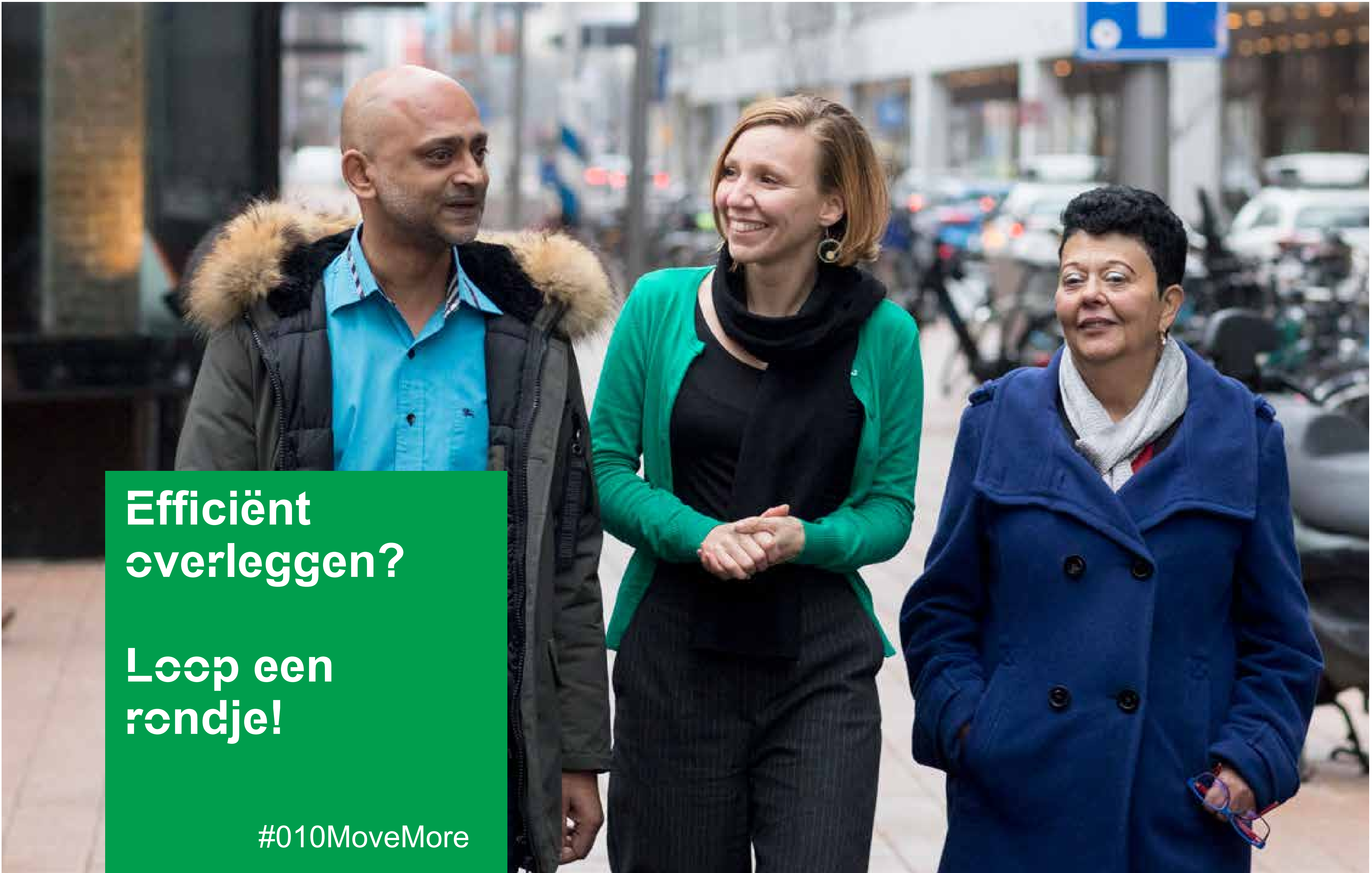

Efficiënt overleggen?

Loop een rondje!

#010MoveMore

Gemeente Rotterdam

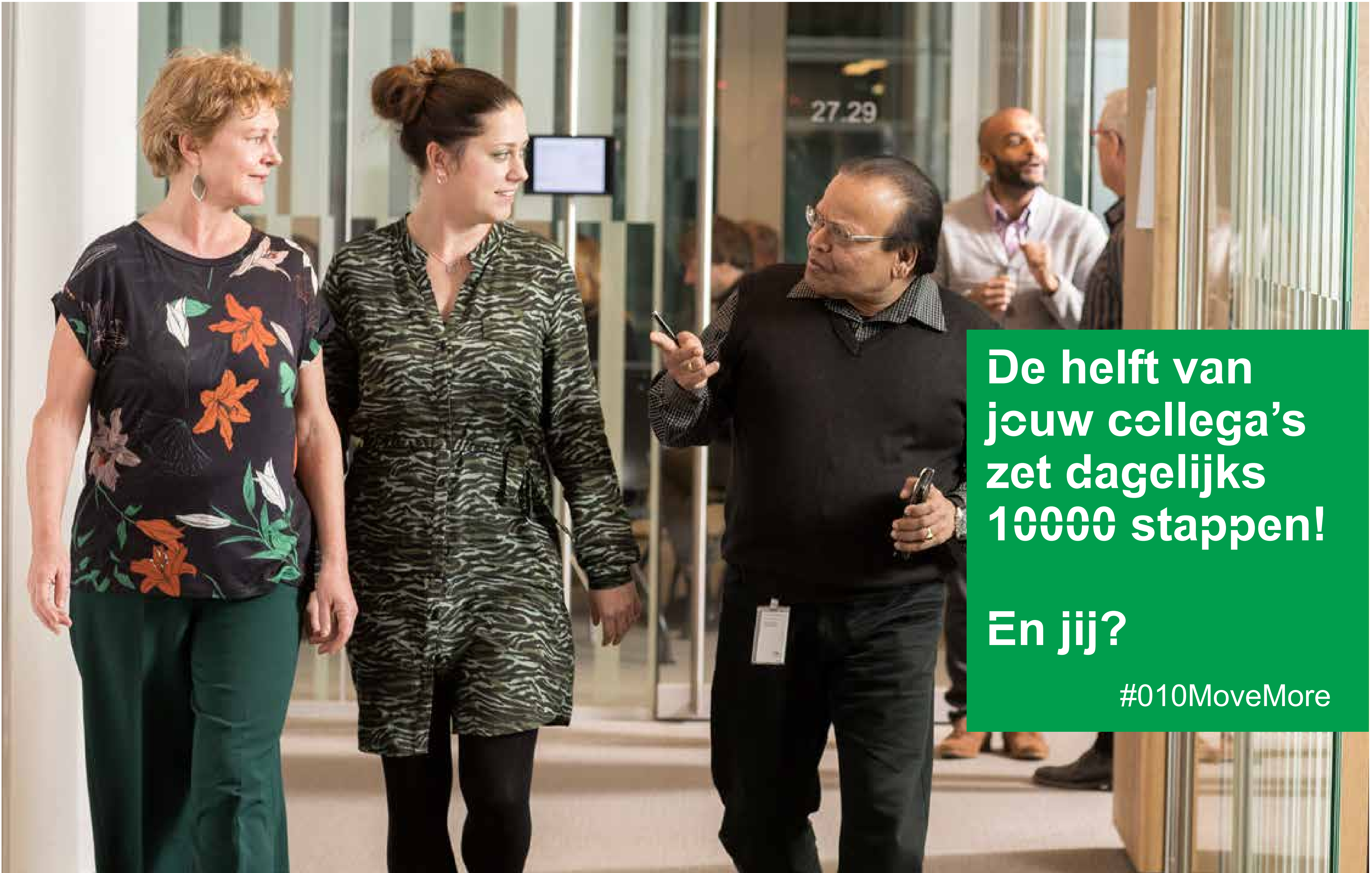

Gemeente Rotterdam

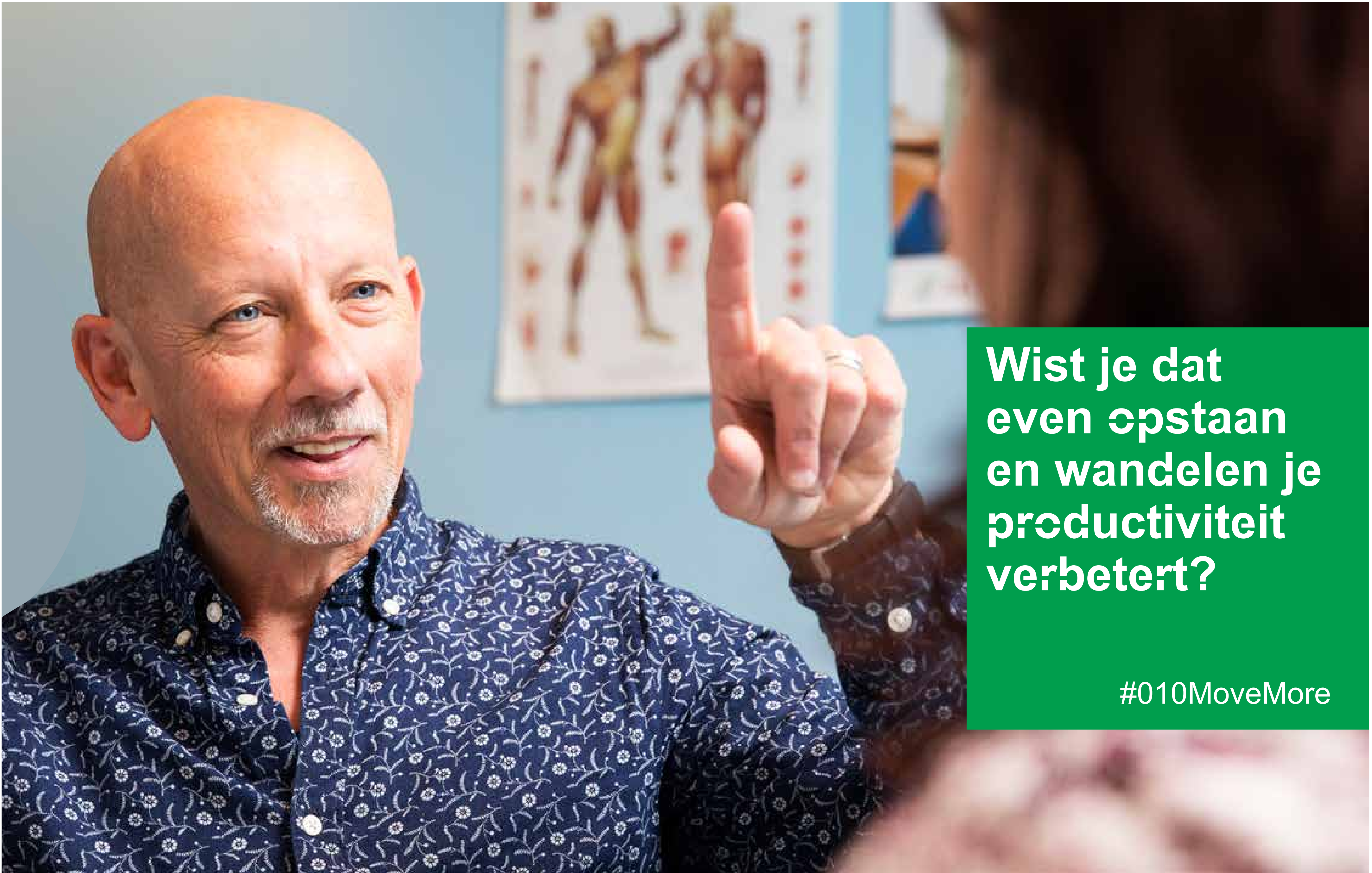

Gemeente Rotterdam

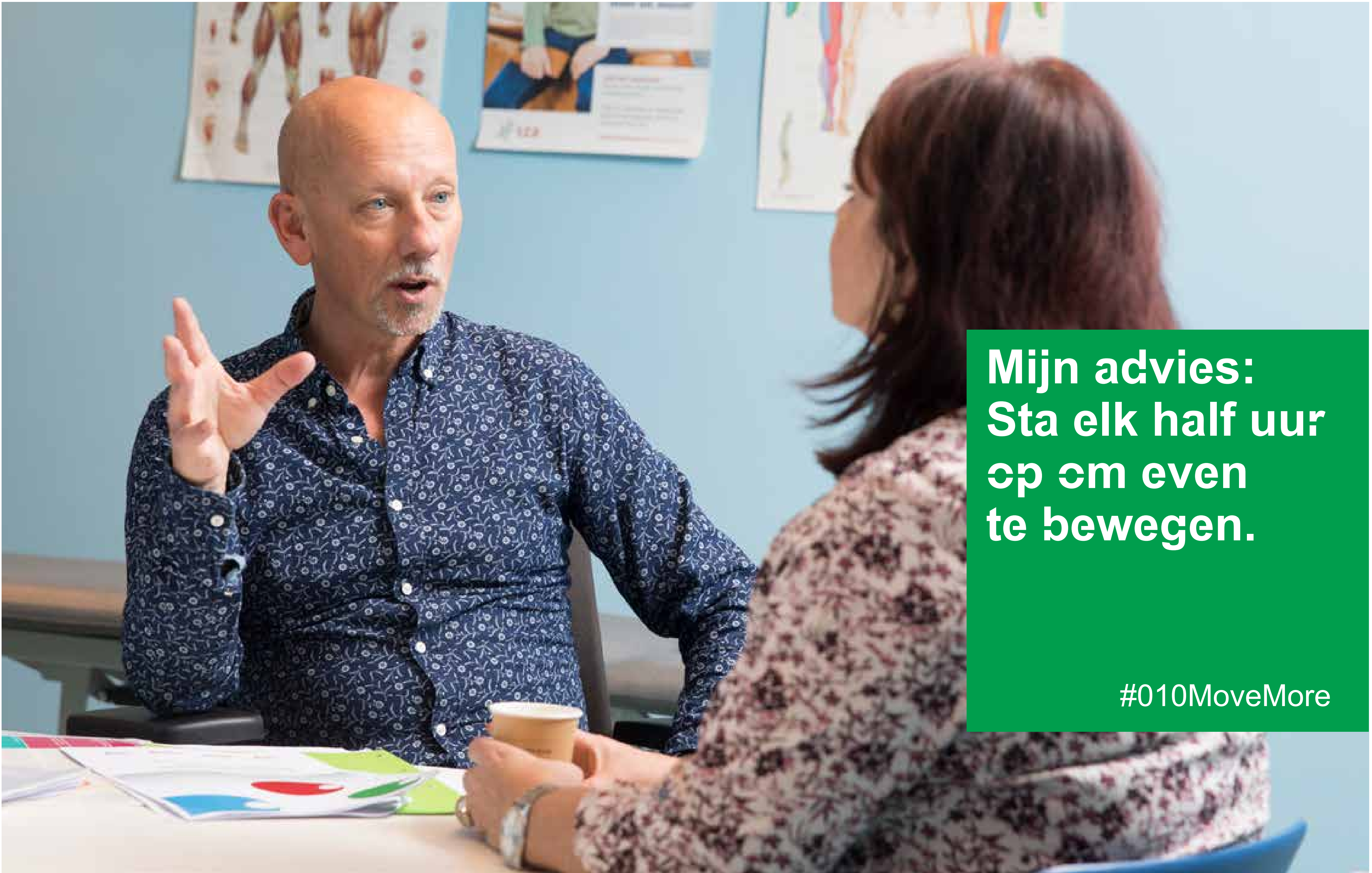

Supplement: Multimedia Appendix 3 [file jmir_v23i4e19875_app3.pdf]
